# Juxtacellular Labeling of Stellate, Disk and Basket Neurons in the Central Nucleus of the Guinea Pig Inferior Colliculus

**DOI:** 10.3389/fncir.2021.721015

**Published:** 2021-11-01

**Authors:** Mark N. Wallace, Trevor M. Shackleton, Zoe Thompson, Alan R. Palmer

**Affiliations:** ^1^Hearing Sciences, Mental Health and Clinical Neurosciences, School of Medicine, University of Nottingham, Nottingham, United Kingdom; ^2^Medical Research Council Institute of Hearing Research, School of Medicine, University of Nottingham, Nottingham, United Kingdom

**Keywords:** microcircuits, inhibitory neurons, basket cells, chopper responses, onset cells, bushy cell, fibrodendritic laminae

## Abstract

We reconstructed the intrinsic axons of 32 neurons in the guinea pig inferior colliculus (IC) following juxtacellular labeling. Biocytin was injected into cells *in vivo*, after first analyzing physiological response properties. Based on axonal morphology there were two classes of neuron: (1) laminar cells (14/32, 44%) with an intrinsic axon and flattened dendrites confined to a single fibrodendritic lamina and (2) translaminar cells (18/32, 56%) with axons that terminated in two or more laminae in the central nucleus (ICc) or the surrounding cortex. There was also one small, low-frequency cell with bushy-like dendrites that was very sensitive to interaural timing differences. The translaminar cells were subdivided into three groups of cells with: (a) stellate dendrites that crossed at least two laminae (8/32, 25%); (b) flattened dendrites confined to one lamina and that had mainly en passant axonal swellings (7/32, 22%) and (c) short, flattened dendrites and axons with distinctive clusters of large terminal boutons in the ICc (3/32, 9%). These terminal clusters were similar to those of cortical basket cells. The 14 laminar cells all had sustained responses apart from one offset response. Almost half the non-basket type translaminar cells (7/15) had onset responses while the others had sustained responses. The basket cells were the only ones to have short-latency (7–9 ms), chopper responses and this distinctive temporal response should allow them to be studied in more detail in future. This is the first description of basket cells in the auditory brainstem, but more work is required to confirm their neurotransmitter and precise post-synaptic targets.

## Introduction

One of the long-term goals in neuroscience has been to identify the function of morphologically and chemically distinct neurons in the central nervous system in the expectation that this would allow us to predict and explain an organism’s behavior, at least in small invertebrates (de Bono and Maricq, 2005). Larger mammalian brains are much more challenging and the progress in identifying clear morphological/functional links in the inferior colliculus (IC) has been rather slow until the recent introduction of new technology for genetic manipulation. The use of Cre transgenic mouse lines is very promising (Chen et al., 2018; Silveira et al., 2020) and has allowed the description of a subset of glutamatergic stellate cells, with spiny dendrites, that contain vasoactive intestinal peptide and have a distinctive set of input/output connections (Goyer et al., 2019). More recently it has been shown that cholecystokinin containing, disk-shaped neurons form 63% of the excitatory central nucleus of the IC (ICc) population and project exclusively to the ventral medial geniculate nucleus (Kreeger et al., 2021). Neuropeptide Y is also a distinctive marker (Nakagawa et al., 1995) and among the GABAergic neurons in the ICc, 38–50% are stellate cells with translaminar dendrites that contain neuropeptide Y (Silveira et al., 2020). Other distinctive molecular markers such as enkephalin (Nakagawa et al., 1995) or nitric oxide synthase (Coote and Rees, 2008; Fujimoto et al., 2016; Olthof et al., 2019) may be of use in defining distinctive neuronal types in future, as may somatostatin or substance P (Wynne et al., 1995; Wynne and Robertson, 1997). Overall, 27% of neurons in the guinea pig IC are GABAergic with less in the dorsal cortex (ICd) and slightly more (30%) in the ICc (Beebe et al., 2016). The proportions in cat (Oliver et al., 1994) and rat (Merchan et al., 2005) are similar and they have been divided into various sub-types based on both intrinsic physiological properties (Ono et al., 2005), size of soma and surrounding molecular markers (Beebe et al., 2016) and the expression of neuropeptide Y (Silveira et al., 2020). The remaining neurons appear to be glutamatergic (Ito et al., 2011) because, although there are many glycinergic synapses in the ICc, the only glycinergic somata in the ICc also appear to be GABAergic (Fredrich et al., 2009). Much more work is required in defining the characteristics of unique neuronal phenotypes. Combined studies of morphology, physiological responses and molecular profile are clearly the way forward (Goyer et al., 2019; Kreeger et al., 2021), but it is difficult to do all three at the same time without selecting one particular subtype (Ito, 2020). In this study we chose to concentrate on fully describing the complete intrinsic axon and making an *in vivo* physiological profile.

In other brainstem auditory nuclei unique relationships have been described between neuronal morphology and the temporal response profiles to pure tone stimuli. In the cochlear nucleus there are seven distinct neuronal types with characteristic physiological properties that are the source of seven parallel pathways that project directly or indirectly to the IC (Cant and Benson, 2003). These include large stellate cells with onset responses, small/medium stellate cells with chopping responses and bushy cells with sustained responses (Rhode et al., 1983; Palmer et al., 2003; Arnott et al., 2004). Large and small stellate cells are also common in the ICc, but we are not aware of any previous description of bushy cells. There are also many cells with onset and chopping responses as well as other types of sustained response (Le Beau et al., 1996; Syka et al., 2000), but they have not yet been linked to a particular morphological type. A number of studies have labeled neurons in the IC by either intracellular (Oliver et al., 1991) or juxtacellular methods (Wallace et al., 2012; Ito, 2020) so that their morphology can be linked to their temporal response profile. Despite this, we still lack a clear framework for linking distinctive morphological features with temporal response profiles to pure tones (Ito, 2020) as has been found in the cochlear nucleus. Part of the problem is that most if not all histological sections have to be stained for the injected marker, in order to locate the labeled soma. Even when double or triple labeling is used only a limited number of molecular markers can be studied and there is a risk that incubating the sections subsequently can lead to the original label either being obscured or some of the signal lost, causing an inability to completely reconstruct the intrinsic axon. Thus, in this study we decided to concentrate on stimulating with a limited number of tonal stimuli and to stain all the sections for biocytin alone. We tried to restrict our injections to the ICc where the cells have strong lemniscal inputs and respond well to pure tone stimuli.

## Materials and Methods

This study describes results from 32 tricolor guinea pigs of either sex weighing from 334 to 972 g (mean 574 ± 161 g) where cells in the IC were visualized by juxtacellular labeling (Pinault, 1996; Arnott et al., 2004). The results for 15 of the cells with laminar axonal and dendritic arborizations have already been published (Wallace et al., 2012). All procedures complied with the United Kingdom Animals (Scientific Procedures) Act 1986 and the EU Directive 2010/63/EU following approval by the Ethical Review Body at the University of Nottingham. All reagents were obtained from Sigma, unless otherwise stated.

### Animal Preparation

Animals were anesthetized with urethane (0.9 g kg^–1^ i.p., in 20% solution in 0.9% saline) and Hypnorm (0.2 ml, i.m., comprising fentanyl citrate 0.315 mg ml^–1^ and fluanisone 10 mg ml^–1^ Vetapharma). Atropine sulfate (0.12 mg, s.c.) was administered at the start of the experiment to reduce fluid secretion in the airways. Anesthesia was supplemented, when necessary, with further doses of Hypnorm (0.2 ml, i.m.). A tracheotomy was performed and the animal was artificially respired with 100% oxygen using a Harvard Apparatus model 970 ventilator. A rectal probe and heating blanket (Harvard Apparatus Homeothermic Blanket Control Unit 50787) were used to keep the body temperature at 38°C. The animal was mounted in a stereotaxic frame where the ear bars were replaced with hollow plastic specula to allow delivery of sound stimuli. Pressure within the middle ear was equalized by a narrow polyethylene tube (0.5 mm external diameter) sealed into the bulla on each side. Brain pulsations were reduced by incising the dura above the posterior fossa to reduce the pressure in the cerebrospinal fluid. The head was leveled so that the surface of the skull in the rostrocaudal axis was horizontal at points 5 and 13 mm in front of ear-bar zero (Rapisarda and Bacchelli, 1977). Bilateral craniotomies were performed, extending 2–3 mm rostral and caudal of the interaural line and 3–4 mm lateral to midline. The dura was removed and the brain covered in 1.5% agar.

### Stimulation, Recording and Juxtacellular Labeling

Stimuli were presented in a sealed system using Radio Shack 40-1377 tweeters (M. Ravicz, Eaton Peabody Laboratory, Boston, MA, United States), with the animals in a sound-attenuating booth, after calibrating the sound levels close to the tympanic membrane. Stimuli were mainly 50 ms tone pips presented to one or both ears at intervals of 200 ms and these were generated using a Tucker-Davis Technologies (TDT) AP2 processor and output at rates of at least 100 kHz using in-line attenuators that limited the output to about 100 dB sound pressure level (SPL).

Units were isolated with aluminosilicate glass capillary microelectrodes (1.0 mm outer diameter with filament, Clarkes SM100F-10, Harvard Apparatus Ltd., Edenbridge, United Kingdom), containing 1.5% biocytin in 0.5 M sodium chloride and a tip impedance of 15–30 MΩ. They were advanced from outside the booth with a piezoelectric motor (Burleigh Inchworm, IW-711-00). Extracellular action potentials were amplified (Axoprobe 1A, Axon Instruments, Burlingame CA), filtered (300–2000 Hz) and discriminated using a level-crossing detector before being recorded with a resolution of 1 μs (TDT SD1 and ET1).

Once a stable unit had been isolated the responses were characterized using a battery of prepared stimuli that included the following: (1) Frequency response areas (FRAs) measured in pseudo-random order by presenting binaural, single tones over a 6 octave by 100 dB range in steps of 1/8 octave and 5 dB. (2) FRAs as above but with a characteristic frequency (CF) tone simultaneously presented at 10 dB above threshold to reveal inhibitory areas in units with low spontaneous activity. (3) Rate-level functions to 10 repetitions of CF tones presented separately to the left and right ears and to both ears. The tones were presented in pseudorandom order over a 100 dB range in 5 dB steps. (4) Peri-stimulus time histograms (PSTHs) of responses evoked by 150 repetitions of CF tones presented to each ear separately and to both. The tones were delivered at 20 dB above the CF response threshold. All tones were gated on and off with a 2 ms rise/fall time with cosine squared windows (TDT Cos2Gate). (5) Interaural level difference (ILD) functions were measured by setting the contralateral tone to 20 dB above CF threshold and varying the level of the ipsilateral tone over a range of ±20 dB above and below that level in 2 dB steps for 10 repetitions. For most units the ipsilateral tone was also set at a constant level and the contralateral tone varied in the same way as above. (6) Interaural time difference (ITD) functions (only when the CF was below about 1.5 kHz) to 20 repetitions of 20 dB suprathreshold CF tones. The ITD was varied in pseudo-random order over 31 steps of 0.1 of the period of the CF tone.

**FIGURE 1:**
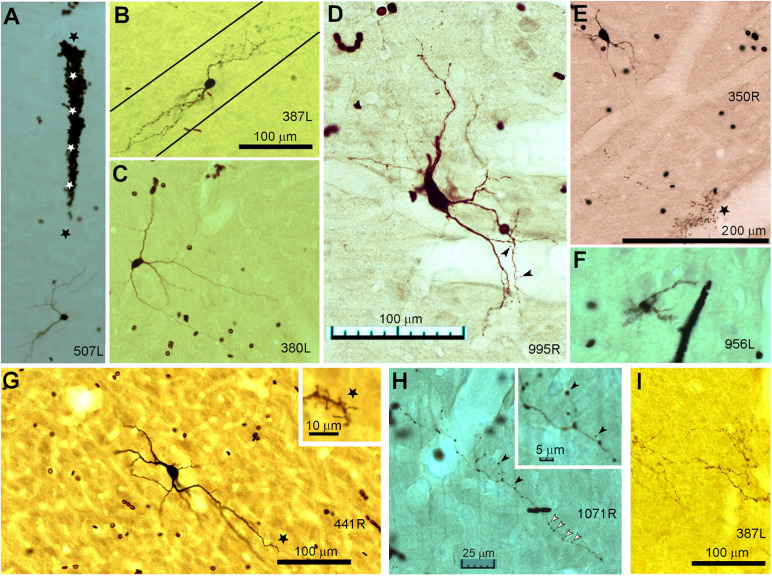
Photomicrographs of biocytin labeled cells in the IC. **(A)** Blood-filled electrode track (marked by stars) directed at the soma of a small laminar disk cell (507L) viewed in the coronal plane. **(B)** Laminar elongated cell (medium size, 387L) with dendrites oriented along a single fibro-dendritic lamina, the orientation of which is shown by the two solid black lines in the coronal plane. **(C)** Medium sized, elongated stellate cell (380L) with dendrites that run across the fibro-dendritic laminae in the coronal plane. **(D)** Large flat disk cell (995R) viewed in the horizontal plane. This has sparse dendritic spines of the pedunculate type which are just visible at this magnification (see arrowheads). **(E)** Medium disk cell (350R, coronal plane) with an axon terminating in clusters of large synapses with a basket-like arrangement one of which is indicated by the black star. **(F)** Small multipolar cell (956L, horizontal plane) with short spiky dendrites and a bushy appearance that lacked an obvious axon. **(G)** Large spherical stellate cell (441R, coronal plane) showing relatively thick dendrites and sparse dendritic spines which are more common at the distal end (see star where the dendrite is shown at higher magnification in the inset). **(H)** Axonal swellings from a small flat laminar cell (1071R, horizontal plane). These can be at the end of short branches and are variable in size (black arrow heads shown at higher magnification in inset) but are more commonly en passant swellings (white arrow heads). **(I)** Examples of mainly en passant axonal swellings for a less flat ovoid cell with an extensive translaminar axon that crosses the commissure (387L). The scale bars for panels **(A–C)** are the same and the scale bar for panel **(D)** also applies to panel **(F)**.

The physiological characterization was completed within 30 min. and if the unit had a stable extracellular spike of at least 1 mV an attempt was made to label it with biocytin according to the juxtacellular method of Pinault (1996). Biocytin was ejected from the recording pipette under physiological control using +3 to +11 nA square wave current pulses of 200 ms duration at 400 ms intervals. Adequate current injection, appropriately close to the cell, caused action potentials to be evoked robustly during the depolarizing epochs. During the current injection, two second long segments of the response were recorded allowing the degree and pattern of driving and the spike waveform to be analyzed *post hoc* (Lin and Liu, 2010). The shapes of the extracellular waveforms were consistent for each unit, but varied greatly between units, with some starting as a positive wave and others as a negative. This may depend on the location of the electrode relative to the axon hillock. In a few experiments the electrode track filled up with blood and showed that the electrode was directed at the soma (Figure 1A), but in other cases the electrode appeared to have contacted the proximal dendrite (Wallace et al., 2012). The speed with which the cell repolarized after the initial depolarization varied between cells and could be conveniently measured by taking the time between the peaks of the positive and negative waves.

The PSTHs were analyzed offline to check for the regularity of spike firing times and this allowed us to determine which cells showed chopping responses (high regularity). Thus, by measuring the time interval between each spike and the one following, an inter-spike interval (ISI) value was assigned to each spike. The PSTHs were plotted with 0.5 ms bins and if there were more than three spikes in a bin then we calculated the mean and standard deviation (SD) for the ISIs assigned to the spikes in that bin. These values were plotted on top of the PSTH. This allowed the regularity of the cell to be measured by calculating an overall coefficient of variation (CV) for the first 40 ms of the response. The CV is the ratio of the SD to the mean ISI (Young et al., 1988; Rees et al., 1997). The sharpness of tuning was analyzed for each unit by calculating the Q_10_ and Q_40_ values from FRAs. The Q_10_ is defined as: CF/bandwidth at 10 dB suprathreshold while the Q_40_ is measured at 40 dB above minimum threshold.

### Histological Reconstructions

Following a survival period of between 2 and 9 h, the animal was given an overdose of pentobarbitone and perfused transcardially with 250 ml 0.1 M phosphate buffer pH 7.4 (PB) followed by 500 ml PB containing 4% paraformaldehyde and 0.5% glutaraldehyde. The brain was removed and stored in the same fixative overnight at 4°C. The following day, the brain was embedded in a mixture of gelatine and egg albumin and serial 50 μm coronal or horizontal sections were cut using a vibratome. The freely floating sections were washed twice in PB and were incubated overnight at 4°C in PB containing 0.3% Triton X-100 and avidin-biotin peroxidase complex (ABC Elite, Vector Laboratories). The sections were washed twice in PB before being incubated for 10 min with 0.05% diaminobenzidine (DAB), 0.005% hydrogen peroxide, 0.0015% nickel ammonium sulfate and 0.0015% cobalt chloride in PB.

Three-dimensional reconstructions were achieved using computer software (Neurolucida, Microbrightfield, Colchester, VT, United States) connected to a microscope (Axioskop2, Carl Zeiss) with a motorized stage. Neuronal processes were traced using a 40× objective lens (NA 0.95) in relation to surface contours for each section. In the two cells with basket-like endings the axonal swellings were all assigned markers in the reconstructions and this allowed a nearest neighbor analysis to be performed with the Neuroexplorer module of the software. Rather than measuring the convex hull volume or performing a Sholl analysis of the dendritic branching pattern as has been done previously (Malmierca et al., 1993, 2011; Ito, 2020) we chose to measure the maximum length of the dendrites within the local lamina and the thickness of the dendrites across the width of the local lamina based on an estimate of the orientation of the lamina at that point. We were unable to discern the precise borders of the laminae or the different divisions of the IC as the sections were not counterstained. We therefore judged the approximate orientation of the laminae based on a deoxyglucose study of isofrequency laminae in the guinea pig (Martin et al., 1988) and the location of the ICc based on comparison with a standard series of guinea pig sections stained for cytochrome oxidase, which were available from our previous study (Liu et al., 2006). Cytochrome oxidase is useful in determining clear borders although they may not always coincide exactly with the borders determined by cytoarchitectonic criteria (Cant and Benson, 2005). Previous studies have indicated that there are transitional zones between the cortical areas and the ICc and that rodents may not have precise borders between different areas (Malmierca et al., 2011). Thus, we mainly relied on the fact that the ICc has a clear tonotopic gradient and could confirm whether or not the CF of the filled cell was consistent with that gradient by reference to the CF of other cells in the recording track.

### Statistical Analysis

The relatively small numbers of cells in each morphological or physiological group and the rather arbitrary criteria for choosing borders between defined groups meant that statistical methods had to be applied with caution when comparing different groups. Initially we plotted the distribution of data, such as soma or dendritic area, for each group and tried to fit linear regression lines and calculate the regression coefficients based on the least squares method. If the data could be fitted to a straight line reasonably well, then the probability that the slope of the line was significantly different from 0 was calculated. When data appeared to have a normal distribution as with first spike latency, then we were able to use a Student’s *t*-test but when there were small numbers (less than 10) in two groups then it was more appropriate to use a Fisher’s exact test.

## Results

### Dendritic Morphology of Cells With Translaminar Axons

In this study we filled a total of 38 cells which had clear responses to pure tone stimulation and where the soma was located in or at the transitional borders of the ICc. Of these, 14 were laminar cells where both the dendrites and intrinsic axon were confined to a fibrodendritic lamina of about 200 μm in thickness as defined previously in the guinea pig (Malmierca et al., 1995; Wallace et al., 2012). There were also 6 cells with unstained axons. This left 18 cells with translaminar axons where the intrinsic axon extended beyond a single fibrodendritic lamina of 200 μm. These had somata with a variety of sizes and dendritic morphologies as illustrated in Figure 1. The dendritic arrangement of the cells with translaminar axons were subdivided into three groups. The largest group was eight cells with stellate dendrites some of which were oriented across the long axis of the fibro-dendritic laminae and cross two or more laminae. An example of this is shown in Figure 1C where there is a medium sized stellate cell viewed in the coronal plane. The other main subgroup with translaminar axons were seven cells with dendrites that were flattened along the axis of the fibro-dendritic laminae. Examples of this are shown in the coronal sections of Figures 1A,B where the approximate orientation of the fibro-dendritic laminae is shown by the solid black lines. When viewed in horizontal sections this type of cell has an ovoid or disk-shaped arrangement of its dendrites (see Figure 1D). The remaining subgroup of cells with translaminar axons were three medium sized flat cells with axons that terminated in distinctive grape-like clusters. One of these clusters is marked by a black star in Figure 1E at a distance of about 300 μm from the soma. The remaining six cells with unstained axons were similar to the laminar or translaminar cells apart from one which had spiky dendrites that were similar to the bushy cells of the cochlear nucleus (Figure 1F). The translaminar cells generally do not have very prominent dendritic spines but there can be clusters of them on the distal dendrites (Figure 1G).

**FIGURE 2:**
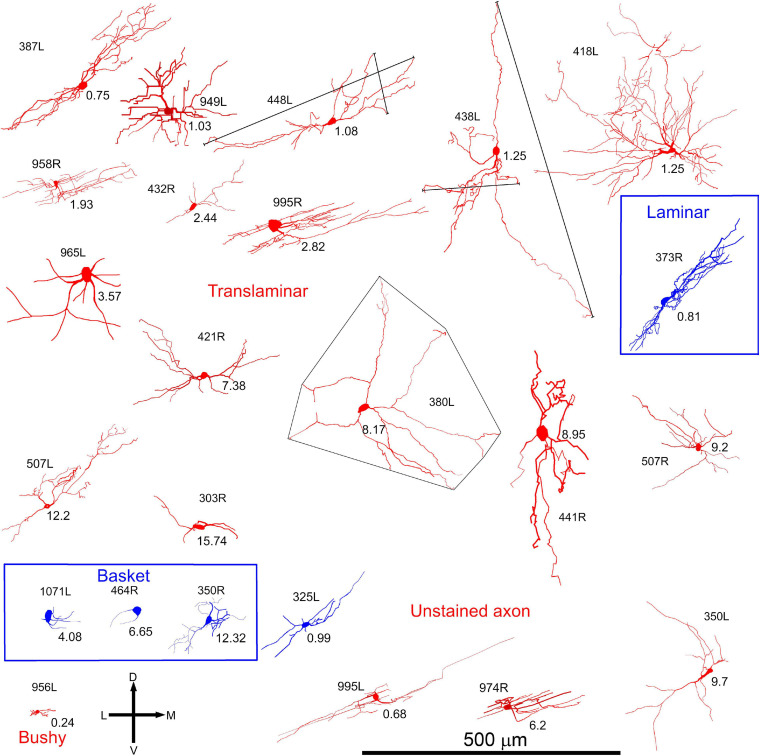
Soma and dendrites of all 24 filled cells viewed in the coronal plane. Most cells were filled in the left IC (L suffix after # number shown to the left or above the soma) but some were filled on the right (R suffix) and these have been reversed so that all cells appear as though they were on the left. The morphology of the dendrites was measured simply by measuring the length between the tips of the two dendrites forming the long axis (see long black lines for cells 448L and 438L) and then the length between the tips of the dendrites forming the short axis (see short black lines for cells 448L and 438L). The area of the dendrites in the coronal plane was taken as that of a polygon drawn around the tips of all the dendrites (see cell 380L). The cells are organized into groups with the largest group (translaminar, 15 red cells) having a variety of dendritic morphologies but a common feature of an axon that has axonal swellings that are not confined to one fibro-dendritic lamina. They are arranged in tonotopic order, from top to bottom, with the CF (in kHz) indicated below and to the right of the soma. Related to this group are the three basket cells (blue cells) where the axon terminates in distinctive clusters of large synapses that appear to form baskets around somata located in two or more nearby fibro-dendritic laminae. The other main group of cells in the central IC is the laminar cells which have both their dendrites and their axons confined to a single fibro-dendritic lamina (one example is shown as the blue cell 373R). Of the remaining cells there were five cells with unstained axons one of which had short bushy dendrites (956L) and one of which gave a short latency chopping response to pure tones that was similar to that of the basket cells (blue cell 325L).

In order to examine the dendrites in more detail all cells were reconstructed with the Neurolucida system and are illustrated in the coronal and horizontal planes. The 18 cells with translaminar axons along with five cells with unstained axons are illustrated in the coronal plane in Figure 2 along with one laminar cell for comparison. This cell was not included in our previous study where we described the other 13 cells with laminar axons and one with an unstained axon (Wallace et al., 2012). The 15 main translaminar cells are shown in red in Figure 2 arranged in a tonotopic sequence with those having the lowest CF placed at the top of the figure and those with a higher CF arranged progressively lower down the figure. Simple measurements were taken from each cell and these included (1) the area of the soma viewed in the plane of section, (2) the area of their dendritic tree encompassed by a polyhedron that joined the tips of their dendrites as illustrated by cell 380L, (3) the number of primary dendrites. We also measured the length of the dendrites from tip to tip along their long axis and the width of their dendritic tree along a line approximately perpendicular to this as illustrated for cells 448L and 438L. These measurements are presented for each cell in Table 1.

**TABLE 1:** Morphological characteristics of the 24 filled cells arranged in four classes with the cells arranged by tonotopic order.

Cell #	CF (kHz)	Soma			Dendrites		Dendritic morphology			Dendrites (μm) min/max. diam.	local axon	projection
		Location	area (μm^2^)	size	area (μm^2^)	number	coronal	horizontal	spines			
**Neurons with translaminar axon**												
387L	0.75	ICc	224	medium	25636	5	less flat	ovoid	medium	90 × 403	ICl & d	commissural
949L	1.03	ICc	174	medium	36588	5	stellate	spherical	sparse	202 × 268	ICc & l	brachium
448L	1.08	ICc	175	medium	37259	3	less flat	disk	–	121 × 442	ICc & l	?
438L	1.25	ICc	198	medium	96296	3	stellate	spherical	–	231 × 648	ICc	?
418L	1.25	ICc	303	large	109434	3	stellate	spherical	–	401 × 412	ICc & l	?
958R	1.93	ICc	180	medium	15592	5	flat	disk	medium	60 × 265	ICc & d	commissural
432R	2.44	ICc	128	small	9875	6	less flat	ovoid	–	102 × 204	ICc	?
995R	2.82	ICc	347	large	24532	5	flat	disk	sparse	60 × 405	ICc & d	?
965L	3.57	ICc	179	medium	33386	6	stellate	spherical	sparse	191 × 232	ICc	?
421R	7.38	ICc	135	small	32320	3	stellate	ovoid	–	198 × 326	ICc	?
380L	8.17	ICc	242	medium	103250	3	stellate	ovoid	–	371 × 459	ICc	brachium
441R	8.95	ICc	308	large	42284	4	stellate	spherical	sparse	158 × 462	ICc	?
507R	9.2	ICc	113	small	26684	5	stellate	ovoid	–	186 × 250	ICc	?
507L	12.2	ICc	105	small	22190	3	less flat	disk	sparse	88 × 324	ICc	?
303R	15.74	ICc	129	small	6759	2	flat	ovoid	sparse	65 × 185	ICc & l	brachium
**Basket cells**												
1071L	4.08	ICc	181	medium	8721	6	flat	disk	–	35 × 144	ICc	none
464R	6.65	ICc	170	medium	1671	2	flat	ovoid	–	30 × 75	ICc	none
350R	12.32	ICc	184	medium	8397	4	flat	disk	sparse	78 × 167	ICc	none
**Laminar neuron**												
373R	0.81	ICc	160	medium	16058	6	flat	disk	–	65 × 350	ICc	?
**Bushy cell (unstained axon)**												
956L	0.24	ICc	53	small	1176	4	bushy	bushy	spikes	28 × 56	ICc	?
**Cells with unstained axons**												
995L	0.68	ICc	267	medium	20433	3	flat	disk	–	48 × 503	?	?
325L	0.99	ICc	130	small	7260	6	flat	disk	–	20 × 141	?	?
974R	6.2	ICc	124	small	10890	6	flat	disk	–	54 × 214	?	?
350L	9.7	ICc	88	small	38974	4	stellate	ovoid	–	182 × 290	?	?

Cells in the IC have often been classified in terms of their soma size and we plotted the size of the soma in all 38 filled cells in ascending order. There were two natural breaks in the progression of ascending size as illustrated in Supplementary Figure 1A. This allowed us to define three natural groups of soma size: small (up to 145 μm^2^, blue), medium (from 146 to 280 μm^2^, red) and large (above 280 μm^2^, green). When soma size was plotted against dendritic area there was a weak trend for the larger somata to be associated with larger dendritic areas. For the group as a whole the regression coefficient was *R*^2^ = 0.14. Even when the regression coefficients were plotted for each of the three groups based on soma size they remained quite low and the slopes were very different, as shown in Supplementary Figure 1B. Thus, it was not possible to accurately predict the dendritic area based on the area of the soma.

**FIGURE 3:**
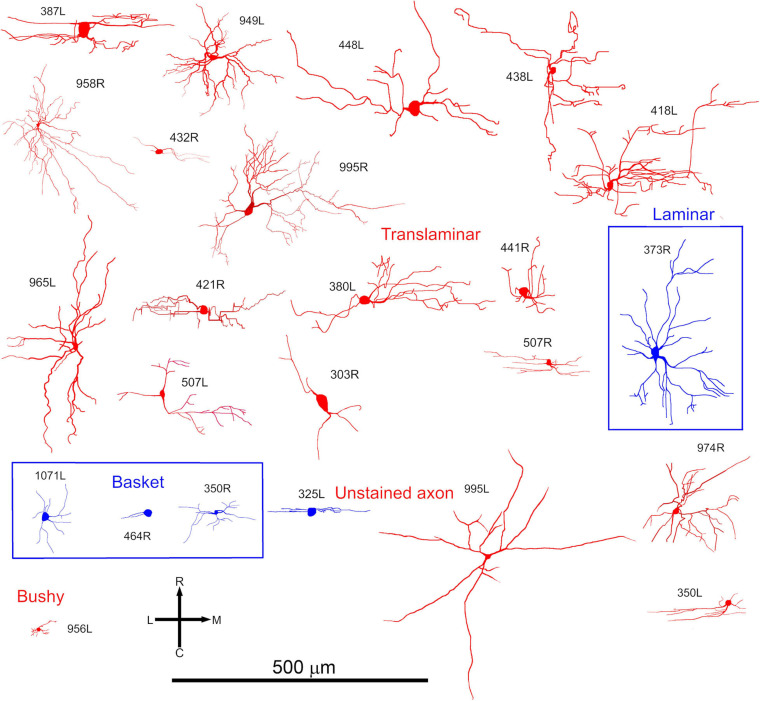
Somata and dendrites of all the cells shown in the same order as in Figure 2 but viewed in the horizontal plane. At this orientation most cells have a stellate arrangement of their dendrites but some have an elongated pattern.

Most of the translaminar cells were multipolar cells and had between 3 and 6 primary dendrites. There were two exceptions of cells with two short, flattened dendrites (303R and 464R), but they did not make a homogenous separate group as one was a basket cell with both dendrites pointing in a similar direction while the other had a much smaller dendritic arbor than any others in the group. When the cells with translaminar axons are viewed in the coronal plane (Figure 2), some have their dendrites flattened and oriented along a fibrodendritic lamina (e.g., cells 387L, 448L, or 995R), while others have dendrites that radiate out in all directions to give a stellate appearance (e.g., cells 418L or 380L). When the same cells are viewed in the horizontal plane (Figure 3) they can also have either a flattened (ovoid) or a stellate appearance. Thus, the translaminar cells can be described in terms of their dendritic morphology in the coronal plane as flattened (flat or less flat) or stellate modified by a term for their shape in the horizontal plane. If they are stellate in both orientations then they are spherical stellate (e.g., 949L, 438L, or 418L), if they are stellate in the horizontal plane but flattened in the coronal then they are flat disk cells (e.g., 995R), if they are stellate in the coronal plane and flattened in the horizontal then they are stellate ovoid (e.g., 421R or 380L) and if they are flattened in both planes then they are flat ovoid (303R and 464R which are unusual in only having two primary dendrites). Each of these four dendritic types can be associated with dendritic trees of different sizes and this is summarized in the diagram in Supplementary Figure 2. Each cell is classified in this way in Table 1.

### Linking Pure Tone Temporal Response Profiles to Cellular Morphology

**FIGURE 4:**
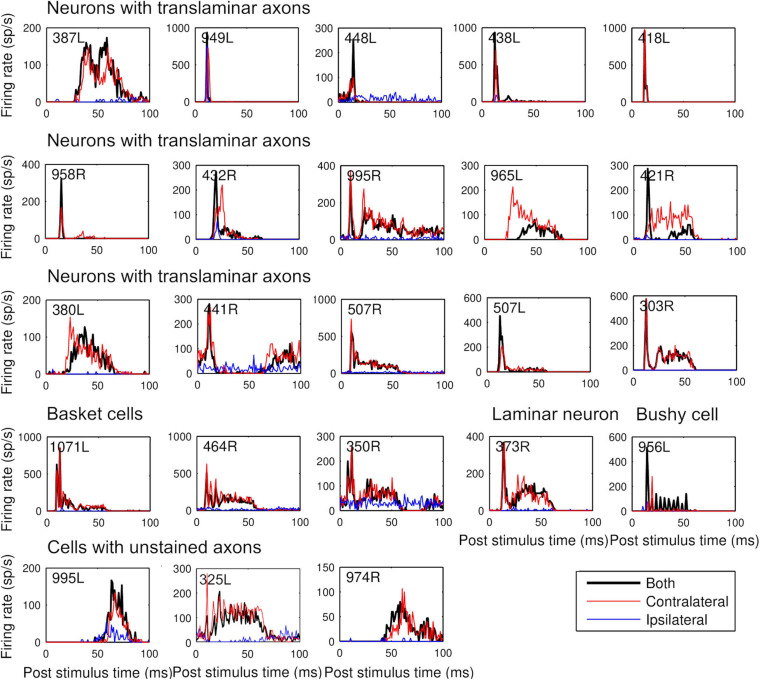
Peristimulus time histograms to 50 ms CF tones arranged, in the same order as Table 1. Tones were presented binaurally (black) contralaterally (red) and ipsilaterally (blue) at 20 dB above minimum threshold. Binwidth is 1 ms.

**FIGURE 5:**
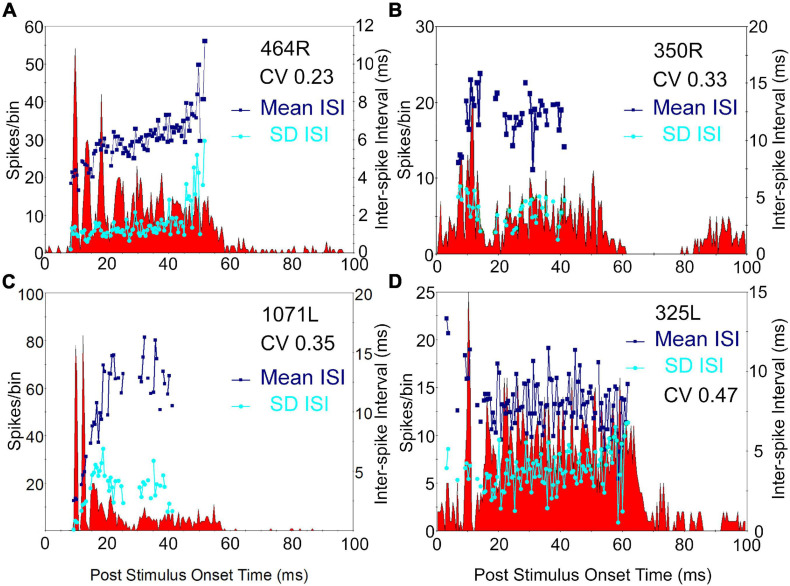
Peri stimulus time histograms (PSTHs) of four cells showing chopper responses. Three of these cells **(A–C)** have basket-like endings and the fourth **(D)** had an axon that was not stained. The histograms show the probability of firing at a set time after the onset of a 50 ms tone presented at 20 dB above threshold at CF, to the contralateral ear. Binwidth is 0.5 ms. A regularity analysis is superimposed on each histogram with the navy blue squares showing the mean interspike intervals (ISI) for the spikes following those in each bin lying between 7 and 47 ms after stimulus onset. The SD of each mean value for a bin is plotted as a cyan dot. These values allow the coefficient of variation (CV) to be calculated and these are indicated in the top right of each panel. For the first three chopping units there are at least two peaks in the firing at the start of the response and these are separated by 3–8 ms.

Our initial studies of morphology indicated that there were three main groups of cells based on the arrangement of intrinsic axons: laminar, translaminar, and basket-like. We looked for any correlation between these groups and the temporal response profile to pure tones. Five main response types have been described in the guinea pig (Le Beau et al., 1996; Syka et al., 2000). These are the onset and chopper and three types of sustained response: on-sustained, sustained without any onset component and pauser types. Examples of each type were found in the current study as illustrated in Figure 4 where the responses to the left and right ear separately and together are shown as PSTHs. The response profile to the contralateral ear was usually dominant and similar to the binaural response. Most of the cells in this study were binaural; only 2 out of the 23 cells showed a monaural response when tones were presented at the same level and phase. The remaining cells all showed either facilitation or inhibition when both ears were stimulated (Table 2). This binaural interaction was more clearly illustrated when rate level functions were recorded and these are shown in Supplementary Figure 3. The temporal profiles were combined with those from our previous study (Wallace et al., 2012) to include more of the laminar cells. This combined population showed two striking results: (1) all of the onset cells had translaminar axons–none of the laminar cells showed onset responses; (2) all of the basket-like cells had short latency chopper responses–the only other cells with chopper responses either did not have a stained axon (325L) or had a longer latency (15 ms; 351L). Chopping responses can either take the form of showing two or more prominent peaks near the start of the PSTH (Figures 5A–C) or a less obvious form with a single peak followed by a regular interspike interval (ISI) (blue dots) with a relatively small SD (cyan dots; Figure 5D). The variation of the SD is used to calculate the CV and in a clear chopper response the CV will be less than 0.3. The cell shown in Figure 5D has more of a sustained response with chopper-like characteristics. Onset cells do not have enough spikes to calculate a CV but where there were enough spikes a CV was calculated and is shown in Table 2. Four of the cells with translaminar axons had CV values of less than 0.4, but they were not considered choppers because they lacked two clear peaks at the start of their response. In this study the three cells with the lowest CV values all had basket-like axonal endings but there were clearly other cells with weaker chopper characteristics (higher CV values) which did not have axons with basket-like endings. We did not fill enough cells to determine where or even if there was a clear cut-off between CV value and basket-like morphology.

**TABLE 2:** Physiological characteristics of the 24 filled cells arranged in 4 classes with the cells arranged by tonotopic order.

Cell #	CF (kHz)	FRA	Inhibitory sidebands		Histograms			Binaural response			Q40	Q10	Regularity (CV)	Dendritic thickness	Lat. (ms)
			Lower	Upper	Contra	Ipsi	Both	Type	ITD	ILD					
**Neurons with translaminar axon**															
387L	0.75	TU	Strong	Strong	Sustained	Spont	Sustained	eO/i	Weak	Monotonic	0.99	2.97	1.08	90	28
949L	1.03	V	Strong	Strong	Onset	Onset	Onset	EE/f	Strong	Monotonic	0.57	1.6	–	268	9
448L	1.08	TD	Weak	Strong	Onset	Spont	Onset	eO/f	None	None	–	–	–	121	12
438L	1.25	V	None	None	Onset	Onset	Onset	Ee/f	None	Monotonic	0.69	2.1	0.93	648	11
418L	1.25	V	Strong	Strong	Onset	None	Onset	Ee/f	None	None	0.89	1.68	–	406	12
958R	1.93	C	Strong	Strong	Onset	None	Onset	eO/F	–	None	1.25	2.12	–	185	14
432R	2.44	V	Strong	Weak	On-sust.	Onset	On-sust.	Ee/i	–	Monotonic	1.17	3.28	0.8	102	15
995R	2.82	V	Strong	None	Pauser	Spont	Pauser	EO/i	–	Monotonic	1.45	6.84	0.39	120	8
965L	3.57	N	Strong	Strong	Sustained	None	Sustained	eO/I	–	Monotonic	10.49	6.25	0.37	212	33
421R	7.38	V	Weak far	Strong close	Sustained	Onset	Pauser	EO/I	–	Monotonic	3.34	6.91	0.33	198	11
380L	8.17	V	Weak	Weak far	Sustained	Spont	Sustained	EO/i	–	Monotonic	1.11	1.89	0.58	459	24
441R	8.95	TD	Strong far	Strong close	Onset	Spont	Onset	eO/m	–	Monotonic	3.54	6.45	0.71	442	10
507R	9.2	V	None	None	On-sust.	Spont	On-sust.	EO/i	–	Monotonic	3.73	7.21	–	250	9
507L	12.2	V	None	Weak	Onset	None	Onset	EO/i	–	Monotonic	2.07	5.6	0.63	88	11
303R	15.74	V	Weak	Weak	Pauser	None	Pauser	EO/m	–	Monotonic	1.29	3.54	0.35	185	11
**Basket cells**															
1071L	4.08	V	Strong	Strong	Chopper	Spont	Chopper	EO/i	–	Monotonic	0.31	22.91	0.35	40	9
464R	6.65	V	None	None	Chopper	Spont	Chopper	Ee/i	–	Monotonic	1.98	6.26	0.23	30	8
350R	12.32	V	Strong	Strong	Chopper	Spont	Chopper	EO/i	–	None	3.54	10.22	0.33	78	7
**Laminar neuron**															
373R	0.81	V	Strong	Strong	Pauser	Spont	Pauser	EO/i	Weak	None	0.9	2.94	0.49	65	12
**Bushy cell (unstained axon)**															
956L	0.24	V	Weak	Strong	Phase-lock	Phase-lock	Phase-lock	eE/i	Strong	Monotonic	–	–	–	28	14
**Cells with unstained axons**															
995L	0.68	C	Strong	Strong	Offset	Offset	Offset	Ee/I	Strong	Monotonic	–	–	–	48	47
325L	0.99	TD	None	None	Chopper	Spont	Chopper	Ei/i	None	Monotonic	1	2.32	0.47	50	10
974R	6.2	V	None	Weak	Offset	Spont	Late/Offset	ee/f	–	Monotonic	1.43	3.84	–	200	43
350L	9.7	V	None	None	–	–	–	–	–	–	0.56	0.78	–	290	15

*FRAs were defined as C: closed, V: v-shaped, TD: tilt down, TU: tilt up, N: narrow. Histograms were defined analogously to those of Le Beau et al. (1996): Sustained units fired throughout the stimulus; Onset units had a brief but strong response at the beginning of the stimulus and few if any spikes thereafter; On-sustained had a clear onset response, but also fired throughout the stimulus; Pausers fired strongly at stimulus onset then paused before firing for the rest of the stimulus; Chopper units showed a regular firing pattern particularly at the beginning of the response; Spont fired at the same spontaneous rate throughout the stimulus; None did not fire before or during the stimulus; Phase-lock units showed regular firing that was locked to the phase of the stimulus; Offset units only responded at the stimulus offset. Binaural responses: three letters show response: contralateral, ipsilateral/binaural: e: weak excitation, E: strong excitation, O: no response, f: weak facilitation, F: strong facilitation, i: weak inhibition, I: strong inhibition (Irvine, 1986).*

Examples of the three types of sustained, non-chopping response were found among both the laminar and translaminar cells. When the first spike latencies were compared across the three morphological groups the mean latencies for the laminar (*n* = 14; 14.9 ± 5.9 ms) and translaminar cells (*n* = 15; 14.5 ± 7.6 ms) were not significantly different from each other (Student’s *t*-Test *p* = 0.45). However, the mean latency for the chopper group (*n* = 5; 9.8 ± 3.1 ms) was significantly different from both the laminar (*p* = 0.02) and translaminar (*p* = 0.03) groups. Within the translaminar group all the onset, on-sustained and pauser cells had latencies of between 8 and 15 ms and in the laminar group all the on-sustained and pauser cells had latencies of 8–15 ms. In each of these groups there were three sustained cells and these all had latencies of 16–33 ms. In the next section long-latency sustained responses were considered to be a separate group from the pauser or on-sustained cells.

### Description of Intrinsic Translaminar Axons in Relation to Binaural PSTH

**FIGURE 6:**
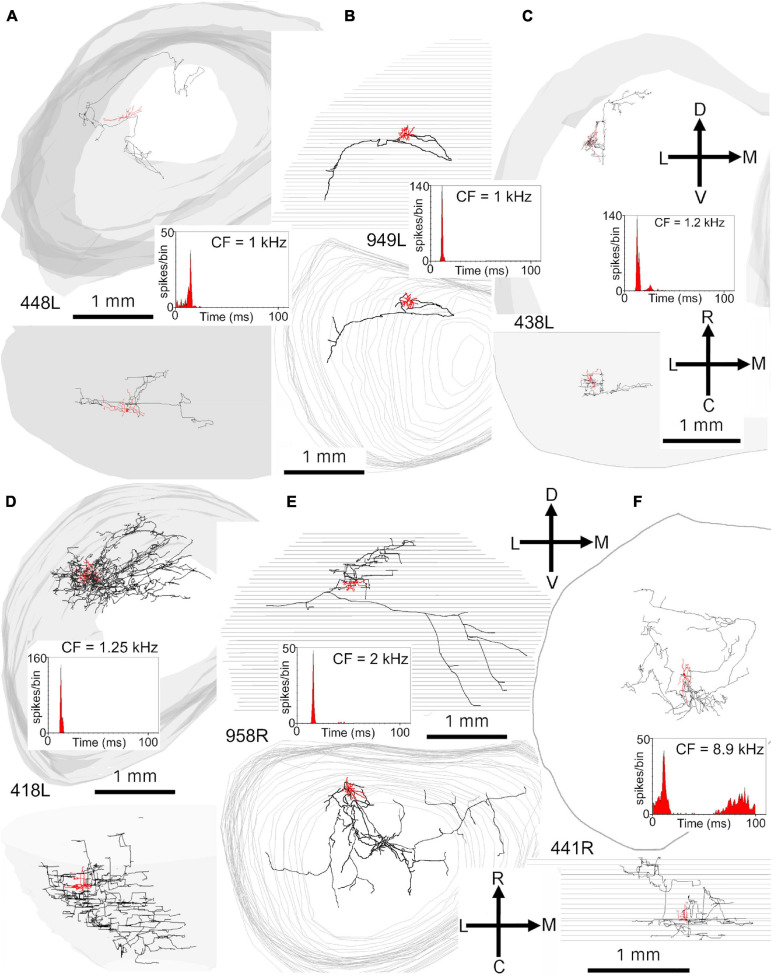
Reconstructions of the axonal ramifications of six onset cells with somata located in the ICc. Soma and dendrites are colored red, the axon is black and the surface contours are gray. Each reconstruction is shown in the coronal and horizontal planes and portrayed as though it was on the left side of the brain, as indicated by the orientation arrows. An inset shows the PSTH in response to a 50 ms long tone pip presented binaurally at 20 dB above threshold at CF. The cells are arranged in tonotopic order. The same description applies to the next three figures as well. **(A)** Disk shaped, laminar (less flat) cell with axonal branches in the LC and ICc that traverse the fibrodendritic laminae. **(B)** Spherical stellate cell located toward the rostral edge of the ICc with a few sparse branches that terminate at two locations in the ICc. **(C)** Spherical stellate cell with long dendrites that extend over at least three laminae and axonal processes within at least two of these laminae. **(D)** Spherical stellate cell located at the lateral edge of the ICc with multiple branches that terminate extensively in the LC and ICc. **(E)** Spiny, disk shaped laminar (less flat) cell located in the rostral part of the ICc, with an axon that mainly terminates in a fibrodendritic lamina, that is dorsal to the soma, but also gives off other branches in traveling toward the commissure. **(F)** Spherical stellate cell, with a large soma located in the rostral part of ICc, with an axon that has extensive branches within ICc.

**FIGURE 7:**
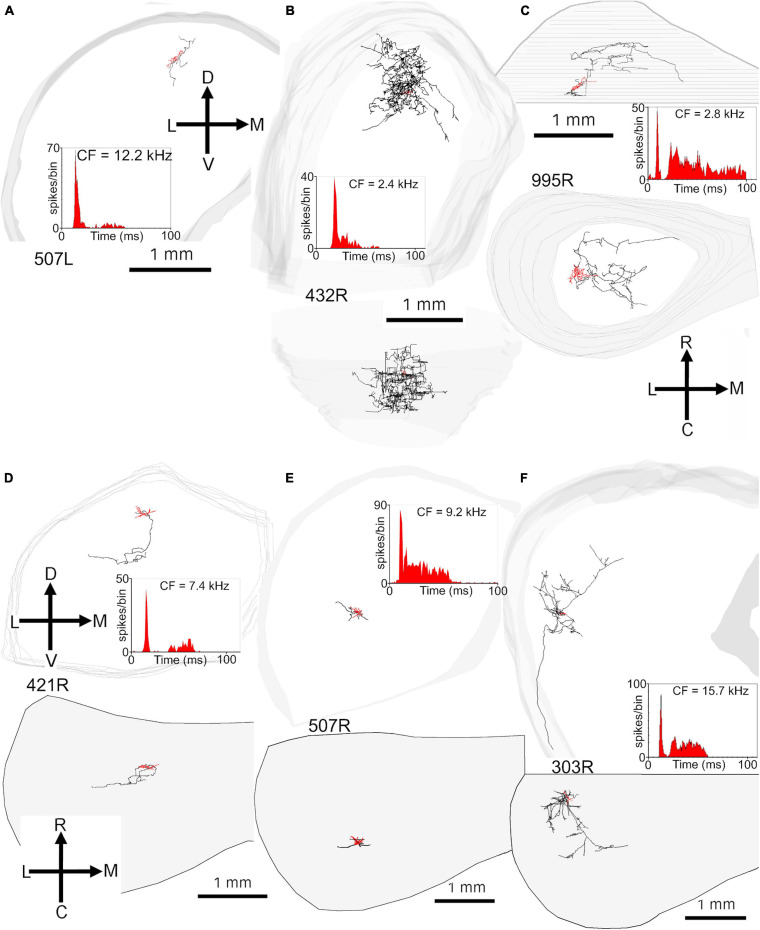
Reconstructions of the axonal ramifications of six onset or on-sustained cells with somata located in the ICc. The cells have varying degrees of pauser-like responses with a reduced response after the onset followed by some degree of recovery. **(A)** Small, less flat disk cell (onset response) with a short, localized axon that contacts two laminae. **(B)** Small ovoid, less flat cell with a dense axonal arbor that stretches across many laminae in the ICc and also into the dorsal cortex. **(C)** Large flat disk cell with dendrites confined to one lamina but with an axon that traverses at least three laminae. **(D)** Small ovoid stellate cell with an axon that terminates in a lamina that is ventral to the one containing the soma. **(E)** Small ovoid stellate cell with a very short axon that is oriented roughly perpendicular to the axis of the laminae. **(F)** Small flat ovoid cell located at the lateral edge of ICc with an axon that terminates in a number of laminae as well as the lateral cortex and then enters the brachium.

Of the 15 translaminar cells, 7 had an onset response and 8 had some sort of sustained response. The cells with an onset response are illustrated in Figure 6 and Figure 7A. They had a range of different dendritic morphologies (Table 1) and intrinsic axons which were mainly confined to ICc, but which may also have contacted the base of the lateral cortex. Their main distinguishing feature is that their axons were not aligned along the fibrodendritic laminae. This is a feature they share with other translaminar cells with a sustained response.

**FIGURE 8:**
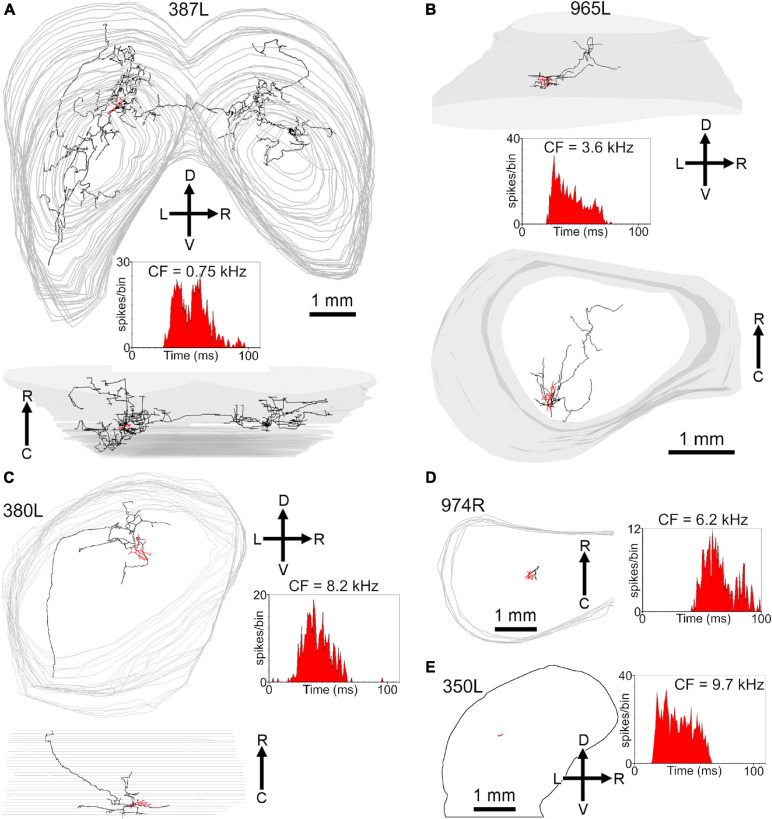
Reconstructions of the axonal ramifications of four long-latency cells and one with an unstained axon, with somata located in the ICc. The first four cells have long latencies of 24–43 ms as indicated in Table 2. **(A)** Medium less-flat, ovoid cell located at the lateral edge of the ICc with an extensive axon that terminates in the lateral and dorsal cortex as well as bilaterally in the ICc. **(B)** Medium spherical stellate located in the ICc with an axon that terminates in the same lamina as the soma but also in others nearby. **(C)** Medium ovoid stellate cell located in the ICc with an axon that has terminal branches in multiple laminae and leaves the ICc via the brachium. **(D)** Small flat disk cell in the middle of ICc with a very short portion of stained axon. **(E)** Small ovoid stellate cell in the ICc with a sustained response (latency 15 ms) and an unstained axon.

The remaining eight translaminar cells had sustained responses: either on-sustained or pauser or else a long-latency sustained response without any onset component (Table 2). The on-sustained and pauser cells are illustrated in Figures 7B–F. They have a variety of dendritic morphologies and their axonal branches have a similar arrangement to the onset cells with all of them crossing the laminae within ICc. There were three cells with sustained responses and all had long latencies (24–33 ms). One less flat ovoid cell (387L; Figure 8A) had extensive axonal branching in the ipsilateral ICc that reached into the lateral and dorsal cortex. It also had a well-filled commissural axon and terminal branches at roughly corresponding points in the contralateral ICc and dorsal and lateral cortices. The two other cells with sustained responses (Figures 8B,C) had axons with more restricted axonal branching within ICc and one (380L) had a long axon that entered the brachium. There were two other cells with sustained responses, but neither had axons that could be recovered for more than a short distance. Both were located in the ICc (Figures 8D,E). The cell in Figure 8D has a latency of 43 ms and mainly responds at the stimulus offset. The only cell that had a pure offset response also only had an axon with a small length that was stained (Supplementary Figure 4A), but again it was located in the ICc. The axonal swellings in the translaminar axons were mainly of the en passant type but some were also on short terminal branches (Figures 1H,I).

Most of the laminar cells were described in a previous report (Wallace et al., 2012), but one was not and is shown for comparative purposes in Supplementary Figure 4B. It was a medium flat disk cell with an axon that branched extensively within the same lamina as the soma was located. The axonal labeling appeared to extend beyond a single lamina, but this was because the lamina was oriented obliquely to the rostro-caudal axis of the horizontal plane and so when viewed in a two-dimensional coronal reconstruction the center of the lamina moved more dorsally at more rostral positions. Within any one histological section the axons were always restricted to a band of less than 200 μm.

### Morphology of Medium Sized Cells With Basket-Like Endings

**FIGURE 9:**
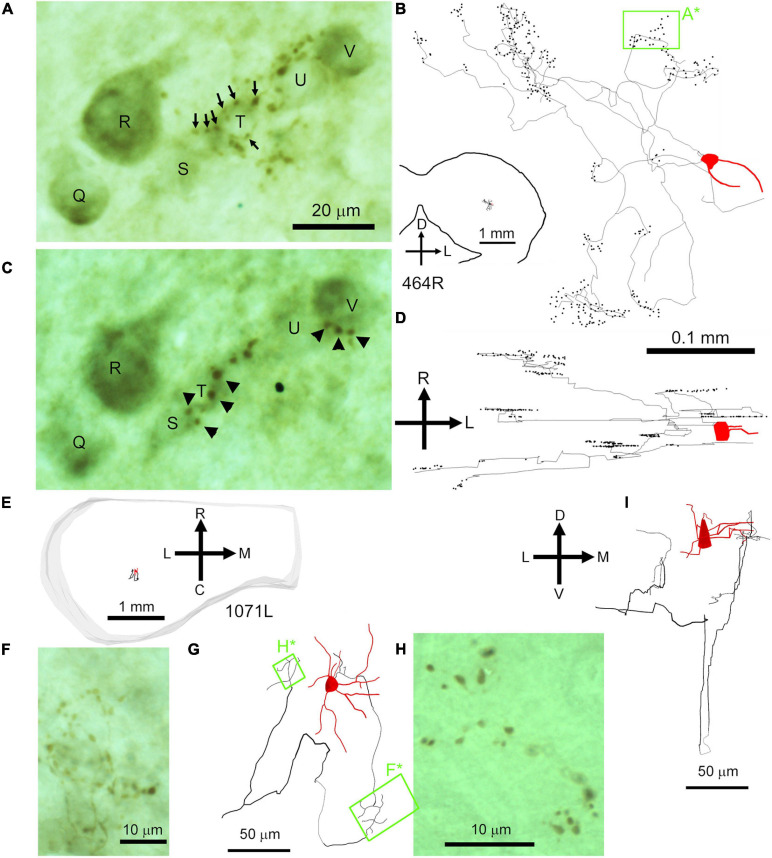
Basket-like axonal endings of two medium flat stellate cells with chopper-like firing responses. The first cell (464R) was located in the central nucleus of the right IC and sectioned in the coronal plane as shown in the inset in panel **(B)**. **(A)** High power view of stained axonal swellings that seem to form baskets surrounding the somata of some nearby cells. These somata can be weakly discerned because of background staining and are labeled Q–V. Some of the swellings associated with soma T are indicated by the small arrows. **(B)** Coronal reconstruction of filled cell with the axonal swellings shown as black dots. The arrows indicate the dorsal (D) and lateral (L) orientations and are positioned over the IVth ventricle. The position of the cells shown in panels **(A,C)** has been indicated by the pale green rectangle (A*). **(C)** The same cells are viewed at a more shallow plane of focus than panel **(A)** to show a different set of axonal swellings, some of which are indicated by arrowheads. Two of the indicated endings seem to be associated with cell S, two with cell T and three with cell V. **(D)** Reconstruction of the same cell shown in the horizontal plane with the rostral (R) direction indicated by the arrow. **(E)** Location of second chopper cell (1071L) in central IC with basket-like axonal endings sectioned in the horizontal plane. **(F)** Basket–like arrangement of faintly stained axonal swellings that may be arranged around three adjacent somata. **(G)** Reconstruction, in the horizontal plane, of the stained cell showing the location of the adjacent panels (F* and H*). **(H)** Photograph of tightly arranged groups of axonal swellings. **(I)** Coronal reconstruction of the filled cell.

Among the translaminar cells there were three with very distinctive clusters of densely packed axonal endings that formed discrete patches relatively close to the soma. The cells were all medium flat cells with a disk or ovoid arrangement of their dendrites that were smooth or sparsely spiny (Table 1). None of the boutons in their clusters was more than 400 μm away from the soma. One of these cells is shown in Figure 9B (cell 464R). In order to avoid obscuring the small axonal swellings the sections were not counterstained, but in this brain the background of non-specific staining was sufficiently dark to identify weakly stained somata. This is shown in Figures 9A,C where the same cells are shown at different planes of focus that are about 5 μm apart. The somata are labeled Q–V and the cells from S–V appear to have stained puncta that are close to their somata. At times these puncta seem to form a net around the soma (e.g., cell T) and are not just “en passant.” There are up to 10 different clusters that form reasonably distinct groups in both the coronal and horizontal (Figure 9D) planes but extending more widely in the medio-lateral direction (340 μm) than in the rostro-caudal direction (125 μm). Individual boutons were identified by markers in Neurolucida to allow a nearest neighbor analysis. A total of 246 boutons were identified and the mean distance to the nearest neighbor was 3.9 μm with a range of 0.9–9 μm. The mean distance between all boutons was 161 μm. This analysis emphasizes how closely packed the axon swellings are and is evidence for specifically targeted endings such as are found with basket cells. The second cell is shown in Figures 9E–I and was only faintly labeled. There were only two clusters of boutons stained and these were within about 100 μm of the soma. The clusters of boutons looked as though they were associated with individual somata, although no counterstain was used to confirm this. As the axon was incompletely filled and there may have been other clusters of axonal swellings. The boutons were not analyzed further.

**FIGURE 10:**
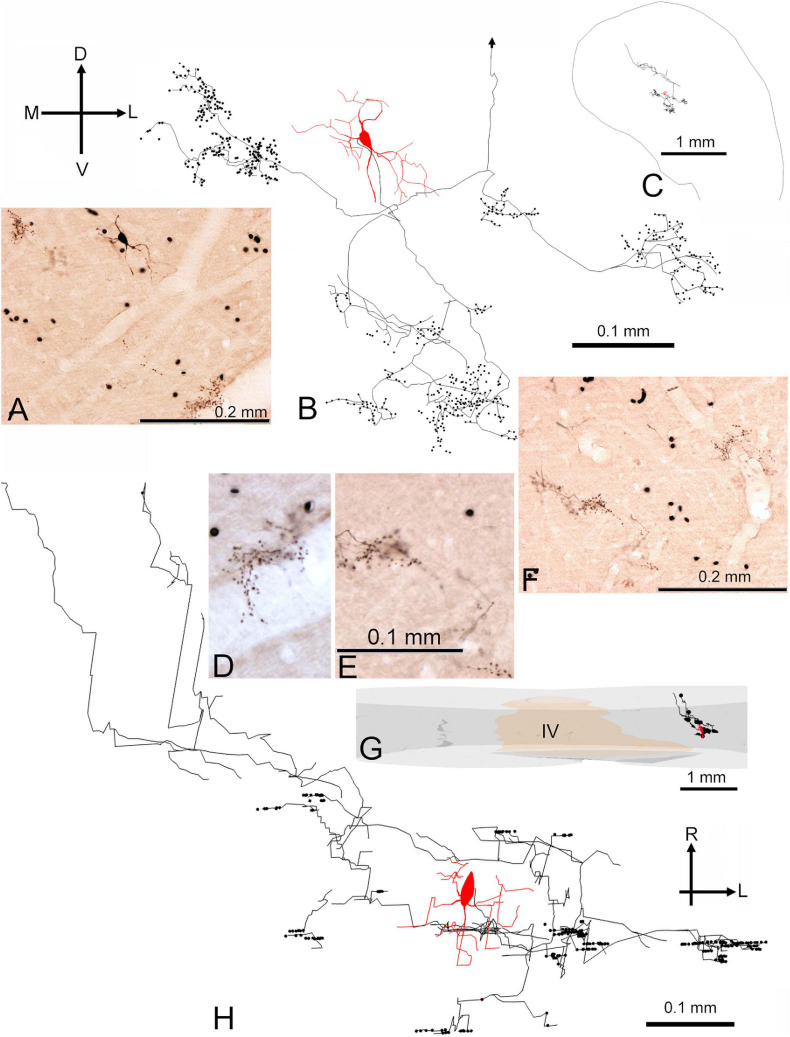
Morphology of medium flat disk cell (350R) with basket–like axonal endings and a chopper response. **(A)** Photograph of stellate soma and the nearby clusters of axonal endings that look like bunches of grapes. **(B)** Coronal reconstruction of the stained cell showing the main clusters of axonal endings in relation to the soma. The dorsal part of the axon is not shown and its truncation point is indicated by an arrow. **(C)** Inset showing the position of the soma and axon in relation to the surface of the IC in a coronal section at the level of the caudal axonal endings. **(D)** Higher-power photograph of clusters of axonal terminals shown at the base of panel **(A)**. **(E)** Higher-power photograph of clusters of axonal terminals shown at the base of panel **(F)**. The scale bar applies to panel **(D)** as well. **(F)** Photograph of three clusters of terminals ventral and lateral to the soma. **(G)** Inset in the horizontal plane showing reconstruction of the cell in relation to the medial borders of the two sides of the inferior colliculus and the IVth ventricle (IV). **(H)** Reconstruction of the complete neuron shown in the horizontal plane.

The third cell is shown in Figures 10A–H (cell 350R) after reconstruction and is displayed in the coronal plane (Figure 10B) and in the horizontal plane (Figure 10H). As with the first cell there are about 10 discrete clusters of different sizes, all with closely spaced groups of axonal swellings. Again, the clusters extend more widely in the medio-lateral direction (550 μm) than in the rostro-caudal direction (300 μm). For this cell we measured 325 boutons and in the nearest neighbor analysis the mean distance was 4.1 μm with a range of 1.3–76.5 μm. The average distance between boutons was 225 μm. Again, this is evidence that the boutons target specific parts of the recipient cells and are not just randomly scattered along the dendrites. It is consistent with a basket cell pattern where the boutons target somata and proximal dendrites. In addition, this cell had an axonal branch that ascended in a rostro-dorsal direction and showed some axonal swellings in a separate lamina. This branch was heading toward the dorsal division of the IC but there was no evidence of the branch terminating there or of it passing through to the commissure.

### Spike Characteristics of Basket Cells

In the neocortex, basket cells form part of the group of sparsely spinous interneurons which are mainly fast-spiking (Kawaguchi and Kubota, 1997). These neurons have a rapid rise time and a rapid repolarization phase which means that they are ready to fire again in about half the time taken by the regular spiking pyramidal cells (McCormick et al., 1985). We wanted to determine if the ICc basket cells were also fast spiking and analyzed their extracellular spike shapes. The extracellular spike shape is more variable than intracellular spikes, but a convenient measure of the membrane channel dynamics is the time between the peaks of the positive and negative waves that form the spike (Lin and Liu, 2010). When the peak-to-trough measurements were made on the three basket cells they were found to have a wide range of spike durations (Supplementary Figure 5) from the fast-spiking cell 1071L (0.39 ± 0.03 ms) to the regular spiking cell 350R (0.99 ± 0.05 ms). The third cell, 464R had an intermediate duration of 0.59 ± 0.03 ms. Thus, there was no evidence of the basket cells forming a single population of fast spiking cells that would allow them to be distinguished from other morphologically defined cell groups in the IC. This is illustrated in the bottom row of Supplementary Figure 5 where a similar range of spike durations is shown for another distinctive group of neurons: the laminar cells with long-latency sustained responses (0.48–1.03 ms). Other details of these cells have already been published (Wallace et al., 2012). Neurons with pauser responses also have a wide range of spike durations as shown by the two examples in the middle row of the Figure where one laminar pauser cell (969L) had a duration of 0.72 ± 0.04 ms while a translaminar pauser (995R) had a duration of 0.32 ± 0.01 ms.

### Relationship of Intrinsic Axons of the Main Neuronal Types to Fibro-Dendritic Laminae

**FIGURE 11:**
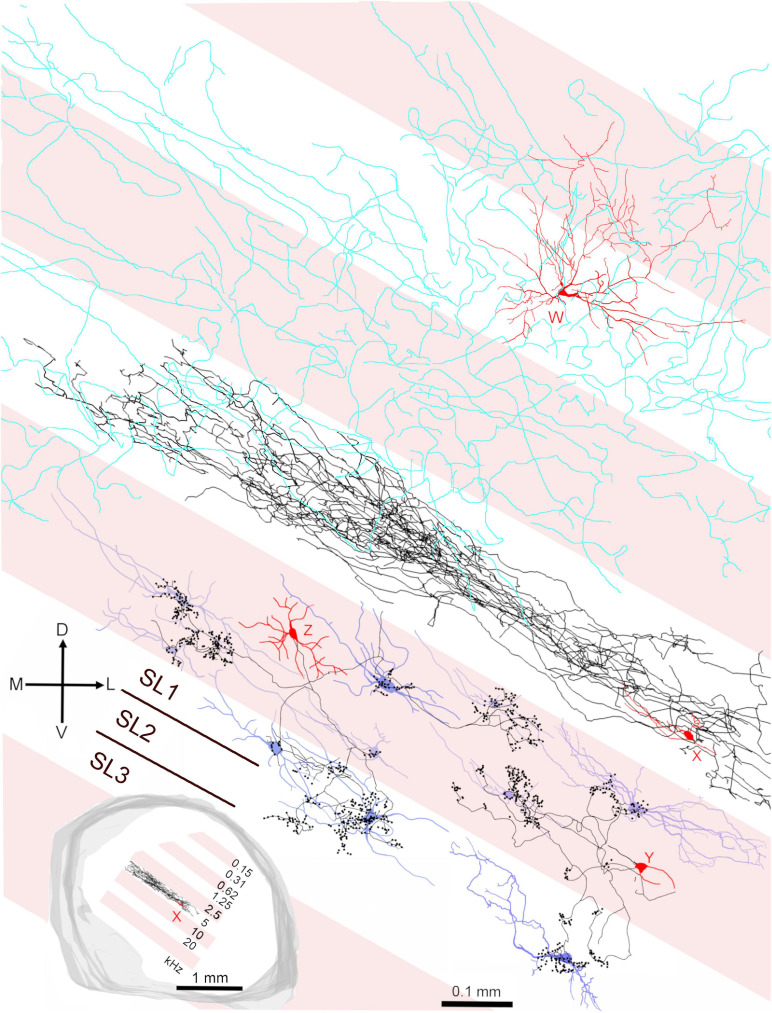
Summary diagram illustrating the relationship of the three main types of cell in the central IC in relation to the fibrodendritic laminae. The alternating pink/white bands represent physiological laminae with an octave spacing as indicated by the inset on the lower left. The cell W (418L) is a large spherical stellate cell (CF 1.25 kHz) with an onset response and an extensive axonal plexus (colored cyan) that crosses several laminae (shown in Figure 8D). The cell X (411L) is a small, flat elongated disk cell (CF 4.9 kHz) with a sustained response and an axonal plexus that is restricted to a single lamina. The cells Y (464R; CF 6.65 kHz) and Z (350R; CF 12.3 kHz) are medium flat cells with chopper responses that have basket-like axonal endings in their own and at least one adjacent lamina. Cells Y and Z show the potential interactions between the axonal clusters of basket cells and laminar disk cells. Each of the disk cells (blue soma and dendrites) was reconstructed in our previous study (Wallace et al., 2012) but have been arbitrarily placed in a lamina irrespective of their actual CF. Each idealized fibrodendritic lamina has a width of 200 μm. Cell Y should be located in the white lamina along with cell X as they have similar CFs. However, it was moved into the adjacent pink lamina for the sake of clarity. The white band centered on 20 kHz has two straight lines in it showing that each lamina may be divided into three sublaminae (SL1-3).

The central nucleus is organized into a series of fibro-dendritic laminae with a thickness of 150–200 μm arranged in a tonotopic sequence (Malmierca et al., 2005; Loftus et al., 2010). Our previous work showed that the distance between the centers of these laminae may represent octave bands at least at mid frequencies and this is shown diagrammatically in the inset of Figure 11 where a single laminar cell (cell X) with its axon is shown in relation to putative laminae with a width of 200 μm. The two cells from Figures 9B, 10B (red somata labeled Y and Z) are shown in relation to these laminae and a selection of laminar cells previously published (Wallace et al., 2012), including cell X, that are used to represent the types of cells that would typically be found in a lamina (blue somata in Figure 11). The axonal clusters of both the basket-like cells have diameters of about 100 μm in the direction of the tonotopic axis. Each cell has at least one cluster in the same lamina as the soma. However, the collective distance of the clusters for each of the two cells is about 300 μm along the tonotopic axis. This implies that some of the clusters are in different laminae. If the laminae are about 200 μm wide, as illustrated, then some of the clusters are clearly in a higher frequency lamina. If the laminae are closer to 100 μm thick then the clusters are in both higher and lower frequency laminae than that of the soma. In either case they seem ideally placed to potentially provide lateral inhibition if it is assumed that they are inhibitory cells. The third type of cell that is illustrated is a spherical stellate cell (red soma, W) with dendrites that cover at least two laminae and whose axon (cyan) may cover up to six laminae.

### Relationship Between Sharpness of Tuning and Dendritic Width

**FIGURE 12:**
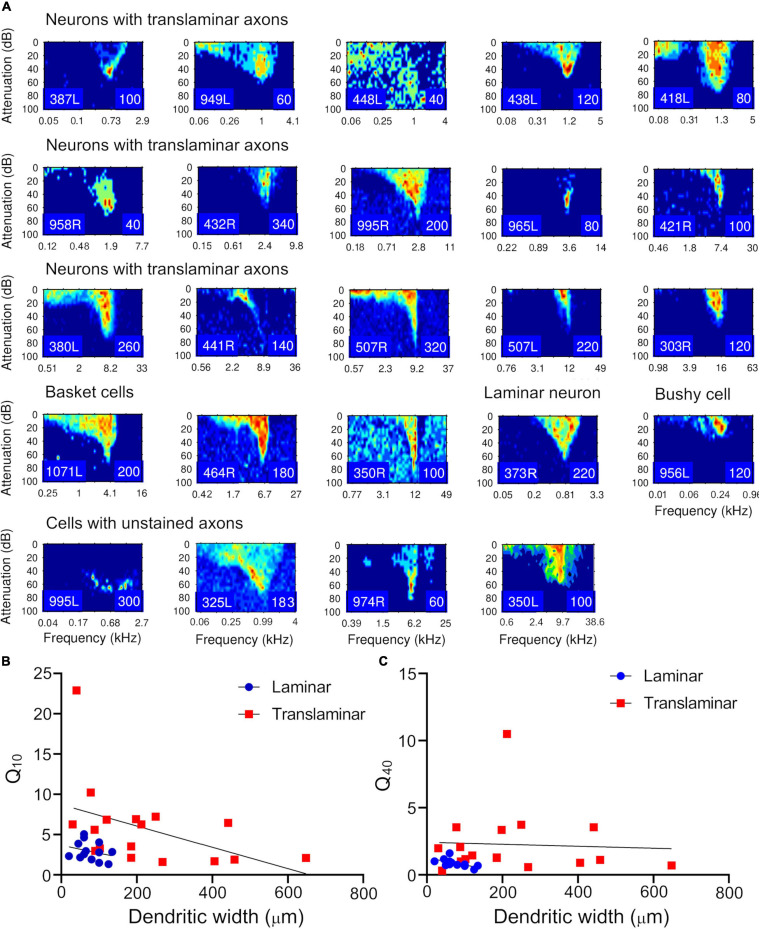
Frequency response areas for the filled cells. **(A)** The arrangement of panels follows the tonotopic order in Table 1 for each class. The response areas are normalized on a temperature scale from blue (zero response) to red (maximum response). The number in the bottom right hand corner of each panel for this and Supplementary Figure 6 indicates the maximum firing rate to which the plot is scaled (spikes/s). **(B)** The relationship between the sharpness of tuning at 10 dB above threshold (Q_10_) and the width of the dendritic tree across the fibrodendritic lamina was plotted separately for the cells with translaminar axons (including the basket cells) in this study (red squares) and the cells with laminar axons from this and our previous study (blue circles). The linear regression line was also plotted for each group. **(C)** The same relationship was also shown by plotting the sharpness of tuning at 40 dB above threshold (Q_40_) for the two groups and again showing the linear regression line.

Frequency response areas were recorded for all filled units as shown in Figure 12A. These corresponded to the different types we have described in the guinea pig previously (Palmer et al., 2013) and are described in Table 2. Most were “V” shaped but other varieties were found in both the translaminar and laminar cells. The sharpness of tuning was assessed at 10 and 40 dB above threshold and the Q_10_ and Q_40_ values for the units are shown in Table 2. This allowed us to plot these values against the width of the dendritic tree along the tonotopic axis to determine if there was a relationship. The Q_10_ data was plotted in Figure 12B for 13 laminar cells (blue circles) and 17 translaminar cells including the basket cells (red squares) and a linear regression is shown for each group separately. For both groups there is a weak trend for cells with flatter dendrites to be more sharply tuned, but neither group can be accurately described by the fitted linear regression line (*R*^2^ values for laminar cells is 0.077 and for the translaminar cells is 0.2). In neither case is the slope of the regression line significantly different from 0 (laminar cells, *F* = 0.92, *p* = 0.36; translaminar cells, *F* = 3.8, *p* = 0.07). The situation is a bit different when Q_40_ values are plotted as shown in Figure 12C. The correlation is much tighter for the laminar cells where the *R*^2^ value is 0.37, *F* = 6.38 and the slope of the regression line is significantly different from 0 (*p* = 0.028). This is not true of the translaminar cells where there is a wide scatter of Q_40_ values and the slope of the regression line is not significantly different from 0: the *R*^2^ value is 0.0028, *F* = 0.04 and *p* = 0.84.

For both the Q_10_ and the Q_40_ values the most striking result is that the range of values is much greater for the translaminar cells than for the laminar cells. This difference can be quantified by splitting the values for each cell type into two groups: Q_10_ < 5 < Q_10_ and Q_40_ < 2 < Q_40_ (Table 3). This forms two 2 × 2 tables which can be conveniently analyzed with a Fisher’s exact test. The probability of the Q_10_ values for the laminar and translaminar cells being from the same populations is 0.0174 and for the Q_40_ values it is 0.0237. This indicates that the translaminar cells are more sharply tuned than the laminar cells. This is despite the fact that the mean width of the laminar cell’s dendrites (*n* = 14; mean = 76 ± 31) is significantly less than the mean width of the translaminar cell’s dendrites (*n* = 18; mean = 218 ± 169) with a probability of *p* = 0.0013 (Student’s *t*-test).

**TABLE 3:** Comparison of numbers of laminar and translaminar cells with broad or sharp tuning.

Numbers of cells	Laminar cells	Translaminar		Laminar cells	Translaminar
Q_10_ < 5	12	8	Q_40_ < 2	13	11
Q_10_ > 5	1	9	Q_40_ > 2	0	6

### Evidence for Inhibitory Sidebands That May Provide a Sharpening of the Frequency Tuning

The fact that the translaminar cells had sharper frequency tuning than the laminar cells, despite having much wider dendritic trees, implies that there may be a functional sharpening of tuning involving some sort of lateral inhibition in the translaminar cells. One way of demonstrating the presence of lateral inhibition is to record two-tone response areas where each tone pip of the stimulation matrix is superimposed on a tone pip that provides constant weak stimulation (tickle tone at 10 dB above threshold at CF). The tickle tone response areas for the filled cells are shown in Supplementary Figure 6 and the presence of areas of inhibition involving either frequencies above or below CF are summarized in Table 2. Among the 18 translaminar cells, including the basket cells, there was evidence of inhibitory sidebands on both sides of CF in 12 cells, on one side of CF in three cells and no evidence of lateral inhibition in three cells. However, there was no obvious correlation between the presence of lateral inhibition and the width of tuning: all 13 laminar cells also showed evidence of lateral inhibition despite having wider tuning than the translaminar cells. It seems to be the closeness of the lateral inhibition to the CF rather than the presence of lateral inhibition *per se* that is involved in determining tuning width.

### Relationship of Cell Type to Interaural Timing Difference (ITD) Sensitivity

Most cells in the ICc are binaural and many are sensitive to interaural differences in either timing or level. The ITD cells are low-frequency and in the guinea pig would be expected to have a CF of less than 1.5 kHz (McAlpine et al., 1996). Of the filled cells with CFs < 1.5 kHz 67% (6/9) of laminar cells and 40% (2/5) of the translaminar cells showed ITD sensitivity. The bushy cell and one cell (325L) with an unstained axon also showed ITD sensitivity and the ITD functions of these 10 cells are shown in Supplementary Figure 7. Half the cells show strong modulation where the firing rate falls close to 0 at some values while the other half show weaker modulation where the firing rate only changes by 10–20% over a full cycle. Most cells (8/10) have a flattened dendritic tree (flat or less flat) or a small bushy shape. There was one cell with a spherical stellate morphology that showed strong modulation (949 L). One common feature of all the cells with strong ITD modulation was that they had short or unstained intrinsic axons. The cells with weak ITD modulation had more extensive intrinsic axons with one having an extensive bilateral plexus that crossed in the commissure (387L) and two with extensive terminal patterns within a single lamina (373R and 339L).

### Relationship of Cell Type to Interaural Level Difference (ILD) Sensitivity

Most binaural cells in the ICc show sensitivity to ILD (Table 2) and among the translaminar cells 12 gave a monotonic response while three were insensitive (Supplementary Figure 8). The bushy cell also gave a monotonic response as did three of the cells with unstained axons. Among the laminar cells 8 gave a monotonic response, 1 gave an off-midline peak response (385R) and five were insensitive. Among the basket cells 2 gave a monotonic response and 1 was insensitive. Thus, there was no clear relationship between cell type and ILD sensitivity.

## Discussion

### Neurotransmitter in Different Morphological Types in the ICc

We have recognized two main morphological types and there are two primary neurotransmitters, but the relationship between them is not simple. In the rodent ICc 99% of neurons are thought to be either GABAergic or glutamatergic (Ito, 2020). The two main morphological types in the guinea pig are: (1) laminar cells (flat and less flat) with both dendrites and axons that are oriented along the characteristic fibrodendritic laminae and (2) translaminar cells that have axons and sometimes dendrites that cross over into two or more fibrodendritic laminae. In the rat the laminar cells often have fairly numerous dendritic spines and many appear to be excitatory (glutamatergic). It is thought that most if not all of the flat laminar cells are glutamatergic (Fredrich et al., 2009; Kreeger et al., 2021) while some of the less flat laminar cells are GABAergic (Ito, 2020). The translaminar cells have comparatively few spines and some are inhibitory [GABAergic; (Ito, 2020; Silveira et al., 2020)]. That may also be true in the guinea pig. Within the ICc, 30% of cells are GABAergic (Beebe et al., 2016) and so even if most of these are translaminar cells at least half of the translaminar cells must be excitatory. In our random sample 56% of the labeled cells (18/32) were translaminar while 44% (14/32) were laminar. The neurons with the largest soma are most likely to be GABAergic, but it is not possible to identify GABAergic cells purely on the size of their soma (Beebe et al., 2016), dendritic dimensions or density of spines as there is too much overlap between cell types (Ito, 2020). All of the GABAergic cells also seem to contain parvalbumin and about a third of these GABAergic neurons may also contain glycine (Fredrich et al., 2009) while others contain nitric oxide synthase (Fujimoto et al., 2016) or neuropeptide Y (Silveira et al., 2020). In our material the dendritic width of flat cells in the shortest dimension was ≤80 μm, while in the less flat cells it was 80–150 μm and the stellate cells had dendritic widths of >150 μm. The translaminar cells had three different types of dendritic tree: flat, less flat and stellate while the laminar cells were either flat or less flat. We were primarily interested in linking dendritic/axonal morphology to physiological properties and were unable to assess neurotransmitter content.

### Relationship Between Intrinsic Processes and Fibro Dendritic Laminae

The ICc is characterized by laminae that reflect the dendritic orientation of the flattened or disk-like cells (Morest, 1964; Rockel and Jones, 1973; Oliver and Morest, 1984) the laminar intrinsic axons (Saldana and Merchan, 1992; Malmierca et al., 1995; Wallace et al., 2012) and the laminar inputs from the lower brainstem (Oliver et al., 1997; Anderson et al., 2006). The terminal fields of axons from the cochlear nucleus may overlap several laminae, especially in the low-frequency (dorsal) zone where they can be up to 700 μm thick (Malmierca et al., 2005). Conversely, in the higher frequency zone, the axonal terminal fields can also be constrained to a single sub-lamina of about 70 μm thick. Despite this large range both the terminal fields of axons from the cochlear nucleus (ventral and dorsal divisions) and the inputs from the lateral superior olive usually have a thickness of 150–200 μm (Shneiderman and Henkel, 1987; Anderson et al., 2006). There is also evidence of anatomically defined sub-laminae based on narrow (85 μm) bands of input from the dorsal nucleus of the lateral lemniscus in the ferret (Henkel et al., 2003) and laminae divided in two in the rat where there are thin laminae of somata with flattened dendrites (∼70 μm wide) adjacent to fibrous interlaminar bands of a similar width (Malmierca et al., 1993). The anatomical laminae have a complex three dimensional arrangement where the orientation and thickness of the fibrous laminae varies depending on the rostro-caudal position (Malmierca et al., 1995). The physical representation in the ICc is not uniform across the whole frequency range when it is considered on an octave scale in guinea pig (Anderson et al., 2006) or monkey (FitzPatrick, 1975) with laminae corresponding to higher frequencies generally having a higher volume. This change in volume is mainly related to the length of the laminae (longer at higher frequencies). There is no indication that the dendritic length or somal volume of neurons changes across the tonotopic axis, but the medio-lateral extent of the laminar axons does seem to be smaller in the low-frequency neurons (Wallace et al., 2012).

Functional laminae in the guinea pig have been clearly demonstrated by 2-deoxyglucose uptake (stimulating with two tones at 1.5 octaves apart) when it was shown that the laminae were oriented at 45° to the horizontal at the caudal pole and then twisted round to being closer to the vertical at the rostral pole (Martin et al., 1988). Functional laminae have also been shown by recording sudden jumps in the CF of units along the tonotopic axis in both the cat (Schreiner and Langner, 1997) and rat (Malmierca et al., 2008). The laminae were about 175 μm wide in the cat and about 140 μm wide in the rat. The functional bands in both species corresponded to neural critical bands with a width of about 0.3 octaves. There has not been any similarly detailed mapping of the frequency tuning of neurons in the ICc of the guinea pig, but the range of CFs recorded in the ICc varied from 50 Hz to 45 kHz (Syka et al., 2000; Palmer et al., 2013). Their range of audible frequencies (10 octaves) is greater than the rat (7.5 octaves) and almost as great as the cat [10.5 octaves; (West, 1985)]. For the range of hearing in the guinea pig (0.045–49 kHz at 60 dB SPL) to be split into critical bands of about 0.33 octaves and fitted into a tonotopic gradient in the ICc of about 2.1 mm (Wallace et al., 2012) this implies that the functional laminae (neural critical bands) would need to be about 70 μm thick. This corresponds roughly to the mean thickness of the flat and less flat cells in the previous (Wallace et al., 2012) and current study where the mean thickness of the 29 flat and less flat cells that were reconstructed was 72.2 ± 29.4 μm. By contrast, the minimum dendritic width of the nine stellate cells in this study was 236 ± 88 μm and this implies they will be able to integrate across at least three critical bands as previously suggested (Ehret and Schreiner, 2005). The presence of these critical bands may require local inhibitory networks to be involved in sharpening the frequency tuning and the best candidate for this are potentially the basket cells. The basket cells in this study all had CFs of between 4 and 13 kHz and our reconstruction indicated that the majority of terminals were probably in the same lamina as the soma, but that between a third and a half of terminals were in an adjacent lamina. The arrangement of the terminals was asymmetric and more likely to be in a lamina with a higher preferred frequency than one with a lower preferred frequency. These laminae are tonotopically arranged and about 200 μm wide in the guinea pig (Wallace et al., 2012) over the mid-frequency range of (∼1–16 kHz) where their centers are about one octave apart (Figure 11 inset). Each may be sub-divided into three sub-laminae (SL1-3) as indicated by the brown lines in the 20 kHz band of Figure 11 and each of these would correspond to a critical band.

### Existence and Functional Roles of Basket Cells

Basket cells have only previously been shown to occur in cortical structures. The terminals of the basket cells in this study were arranged in dense clusters in adjacent laminae as well as in the lamina of origin and were generally within 300 μm of the soma. The three basket cells were all medium, flat disk or ovoid cells with thin dendrites, few if any spines and were all located in ICc. They all showed evidence of a chopping response with regular spikes produced in response to pure tones and a short first-spike latency of 7–9 ms. These results suggest that basket cells may make up as much as 9% of the cellular population in ICc and it may be possible to identify them by their short latency, chopping response. However, the small number of basket cells in this study means that we have to be very cautious about making general statements about them. Further work will be needed both in guinea pig and in other species before any confident statements can be made about their role or significance.

We could not find any evidence that interneurons in any part of the IC, with axons ending in pericellular baskets, have been described previously. The presence of peri-neuronal nests of terminal endings around the somata of cells in the ICc had already been described by Cajal in 1911 (Ramon y Cajal, 1995). However, he thought that these nests were formed by ascending fibers from the lateral lemniscus and explicitly said he did not believe there were any interneurons that contributed to these pericellular nests. Confirmation of pericellular nests formed by lemniscal fibers was obtained subsequently (Oliver and Morest, 1984) and a later intracellular study of neurons in the IC (Oliver et al., 1991) again confirmed the belief that intrinsic axons did not form any pericellular baskets in the IC. This is still the current position (Ito et al., 2016; Ito, 2020). Putative inhibitory synapses have been described on the somata of medium and large cells in the ICc (Roberts and Ribak, 1987; Nakamoto et al., 2014; Beebe et al., 2016). Basket cells in both the cerebral and cerebellar cortices are inhibitory and provide a mechanism for binding together the activity of groups of projection neurons by regulating their firing patterns (Cobb et al., 1995). If the basket cells in the IC are also inhibitory then they may fulfill a similar function in the auditory system. The IC may be the first nucleus in the ascending auditory pathway where auditory objects are formed (Nelken, 2008) and so there is a need for local inhibitory neurons that can fulfill a role of binding the activity of disparate neurons together. Intrinsic axons are now recognized as an important source of inhibition in the IC (Sturm et al., 2014), but future work will be required to determine whether or not the basket cells are inhibitory and confirm that they form axosomatic synapses. In the cerebral cortex parvalbumin containing basket cells are fast-spiking while the cholecystokinin containing basket cells are regular-spiking (Armstrong and Soltesz, 2012). The fact that two of the filled basket cells had relatively thin (fast) and one had regular (slow) spikes suggests that the basket cell population in the IC is not uniform. In the mouse IC the cholecystokinin containing, disk-shaped neurons also have a wide range of action potential half-width (from 0.2 to 0.7 ms; Kreeger et al., 2021) and it appears that action potential width is not a distinguishing feature for particular neuronal types in the IC. The three basket cells in this study could all be classified as medium-sized (Beebe et al., 2016) and did not fall into the small and large classes defined in the neocortex (Wang et al., 2002).

We were not able to provide any direct evidence that the basket cells are inhibitory but it would be expected from comparison with other brain areas. Basket cells in the cerebellum, hippocampus and neocortex are all inhibitory (Andersen et al., 1963a,b; Jones and Hendry, 1984; Ribak, 1992). Antibodies for glycine have also shown that it is present in some neurons of the IC (Fredrich et al., 2009) and that it may be confined to the central nucleus (Buentello et al., 2015), but that most glycinergic endings are in the neuropil, not on somata (Merchan et al., 2005). We were not able to use double labeling in the current study because we wanted a permanent stain that would not fade with time and we did not want to risk obscuring the fine axonal endings with a second stain. GABAergic synapses are found on the somata of neurons of all sizes in the IC (Roberts and Ribak, 1987; Oliver et al., 1994) and some of these may arise from the basket cells. The termination of basket endings is highly likely axosomatic. Axosomatic terminals can be glutamatergic (on large GABAergic neurons) or GABAergic (on all cell types). However, basket terminals are unlikely glutamatergic, because single axon branches that make axosomatic glutamatergic terminals on large GABAergic neurons only make 1–6 contacts on a single large GABAergic soma (Ito and Oliver, 2014; Ito et al., 2015), while basket terminals are likely to make many contacts on a single soma. Thus, although there is circumstantial evidence that the basket cells are probably GABAergic this will need to be confirmed directly in future.

Basket cells specifically terminate on the soma of target cells where inhibition would have a powerful action in blocking spike generation (Fishell and Rudy, 2011). There are large numbers of intrinsic axons that could activate the basket cells of the IC (Saldana and Merchan, 1992; Malmierca et al., 1995), but they did not seem to be the main input for the basket cells we studied. When 15 laminar cells with intrinsic axons were characterized previously (Wallace et al., 2012) only one had a minimum first spike latency of less than 10 ms for tonal stimulation and this cell had a high threshold. By contrast all the basket cells had first spike latencies of less than 10 ms. A more likely input for the basket cells is a direct afferent input from the cochlear nucleus. All three cells had a primarily monaural input from the contralateral ear except at the highest sound levels. Having a short latency input direct from the cochlear nucleus would mean that the basket cells could be involved in feed-forward inhibition if they are, indeed, inhibitory. It is already known that local GABAergic inhibition is responsible for sharpening the tuning curve for IC cells in the guinea pig (LeBeau et al., 2001) and basket cells might contribute to this process as their projection to either side of a fibrodendritic lamina would make them ideally suited to sharpening the frequency tuning of projection neurons. The IC shows selectivity for con-specific communication calls and particularly for frequency modulated sweeps. By having an asymmetric arrangement to their groups of terminal boutons the basket cells might be involved in determining if surrounding cells were sensitive to upward or downward FM sweeps (Pollak, 2013).

### Can Different Neuronal Types Be Identified by Their Response Profile?

We originally started this study in the hope that we would be able to find morphological/functional correlations of the sort found in the ventral cochlear nucleus (Rhode et al., 1983; Palmer et al., 2003) despite the lack of clear evidence in the past (Oliver et al., 1991; Peruzzi et al., 2000). We have now described some promising correlations. All the laminar cells had sustained responses while some of the translaminar cells had onset responses. In the rat cortical IC the majority of neurons have onset responses (Perez-Gonzalez et al., 2005) whereas in the guinea pig there were equal numbers of onset neurons in the ICc and IC dorsal (about 25%) and even in IC lateral only about 35% of responses were of the onset type (Syka et al., 2000). Among low-frequency cells those showing strong ITD sensitivity make a separate sub-population, but the only common feature seemed to be the absence of any extensive axon collaterals. These cells may mainly be relay cells that project to the medial geniculate body and form the basis of ITD sensitive columns in the primary auditory cortex (Wallace and Palmer, 2009). Although there are maps of auditory space present in the superior colliculus (King and Palmer, 1983) and lateral cortex of the IC (ICl) (Binns et al., 1992) these seem to be mainly based on high-frequency inputs and would not be expected to receive input from these low-frequency cells. The most distinctive ITD sensitive cell was the small bushy cell. Bushy cells like this have apparently not been described before, possibly because they would look more like glial cells than neurons in a Golgi preparation. In a recent study combining juxtacellular labeling with immunohistochemical labeling it was concluded that in most cases the identification of distinct neuronal types will require the description of a number of parameters (Ito, 2020) but the presence of distinctive neuropeptides may be the most useful (Goyer et al., 2019; Silveira et al., 2020; Kreeger et al., 2021).

A more promising candidate for primarily physiological identification is the basket cell. All three basket cells showed a chopping response to contralateral tonal stimulation, a primarily monaural response and a very short first-spike response latency. As only three basket cells were filled in one species it would be unwise to make any strong assertions about how general these findings could be or state that all basket cells in the IC have chopping responses. There are two main types of chopping response in the IC (Rees et al., 1997): choppers of the type we have shown to be basket cells and pauser-choppers, one of which we filled and found to be a laminar cell (Wallace et al., 2012). The choppers in urethane anesthetized guinea pigs made up 11% of a random population of units in the IC (Rees et al., 1997) and this is not very different to the number of choppers that were filled in this study (3/36; 8%). The main types of cell in the ventral cochlear nucleus that project to the IC (Oliver, 1987) may be either transient or sustained choppers and it would be useful to know if their chopping responses are related to those of the basket cells. Basket cells are clearly an important type of interneuron in the ICc and much more needs to be learnt about them.

## Data Availability Statement

The original contributions presented in the study are included in the article/Supplementary Material, further inquiries can be directed to http://neuromorpho.org/dableFiles/wallace_palmer/Supplementary/Wallace_Palmer.zip.

## Ethics Statement

The animal study was reviewed and approved by Animal Welfare and Ethical Review Board of the University of Nottingham.

## Author Contributions

AP conceived and designed the study. AP and TS collected and analyzed the physiological data. MW and ZT performed the histological process. MW, ZT, and AP made the computer reconstructions of filled cells. MW prepared the figures and wrote the manuscript with the help of AP. All authors approved the final version.

## Conflict of Interest

The authors declare that the research was conducted in the absence of any commercial or financial relationships that could be construed as a potential conflict of interest.

## Publisher’s Note

All claims expressed in this article are solely those of the authors and do not necessarily represent those of their affiliated organizations, or those of the publisher, the editors and the reviewers. Any product that may be evaluated in this article, or claim that may be made by its manufacturer, is not guaranteed or endorsed by the publisher.

## Abbreviations

CF: characteristic frequency
CV: coefficient of variation
D-V: dorso-ventral
FRA: frequency response area
IC: inferior colliculus
ICc: central nucleus of the IC
ICd: dorsal cortex of the IC
ICl: lateral cortex of the IC
ILD: interaural level difference
ISI: inter-spike interval
ITD: interaural time difference
M-L: medio-lateral
PB: 0.1 M phosphate buffer pH 7.4
PSTH: peri-stimulus time histogram
R-C: rostro-caudal
SD: standard deviation
SPL: sound pressure level.

## References

[B1] Andersen P. Eccles J. Voorhoeve P. E. (1963a). Inhibitory synapses on somas of Purkinje cells in the cerebellum. *Nature* 199 655–656. 10.1038/199655a0 14074549

[B2] Andersen P. Eccles J. C. Loyning Y. (1963b). Recurrent inhibition in the hippocampus with identification of the inhibitory cell and its synapses. *Nature* 198 540–542. 10.1038/198540a0 14012800

[B3] Anderson L. A. Malmierca M. S. Wallace M. N. Palmer A. R. (2006). Evidence for a direct, short latency projection from the dorsal cochlear nucleus to the auditory thalamus in the guinea pig. *Eur. J. Neurosci.* 24 491–498. 10.1111/j.1460-9568.2006.04930.x 16836634

[B4] Armstrong C. Soltesz I. (2012). Basket cell dichotomy in microcircuit function. *J. Physiol.* 590 683–694. 10.1113/jphysiol.2011.223669 22199164 PMC3381302

[B5] Arnott R. H. Wallace M. N. Shackleton T. M. Palmer A. R. (2004). Onset neurones in the anteroventral cochlear nucleus project to the dorsal cochlear nucleus. *J. Assoc. Res. Otolaryngol.* 5 153–170. 10.1007/s10162-003-4036-8 15357418 PMC2538402

[B6] Beebe N. L. Young J. W. Mellott J. G. Schofield B. R. (2016). Extracellular molecular markers and soma size of inhibitory neurons: evidence for four subtypes of GABAergic cells in the Inferior Colliculus. *J. Neurosci.* 36 3988–3999. 10.1523/Jneurosci.0217-16.2016 27053206 PMC4821910

[B7] Binns K. E. Grant S. Withington D. J. Keating M. J. (1992). A topographic representation of auditory space in the external nucleus of the inferior Colliculus of the guinea-pig. *Brain Res.* 589 231–242. 10.1016/0006-8993(92)91282-j 1393591

[B8] Buentello D. C. Bishop D. C. Oliver D. L. (2015). Differential distribution of GABA and Glycine terminals in the inferior Colliculus of rat and mouse. *J. Comp. Neurol.* 523 2683–2697. 10.1002/cne.23810 25976159 PMC4607567

[B9] Cant N. B. Benson C. G. (2003). Parallel auditory pathways: projection patterns of the different neuronal populations in the dorsal and ventral cochlear nuclei. *Brain Res. Bull.* 60 457–474. 10.1016/s0361-9230(03)00050-9 12787867

[B10] Cant N. B. Benson C. G. (2005). An atlas of the inferior colliculus of the gerbil in three dimensions. *Hear. Res.* 206 12–27. 10.1016/j.heares.2005.02.014 16080995

[B11] Chen C. Cheng M. Ito T. Song S. (2018). Neuronal organization in the inferior Colliculus revisited with cell-type-dependent monosynaptic tracing. *J. Neurosci.* 38 3318–3332. 10.1523/JNEUROSCI.2173-17.2018 29483283 PMC6596054

[B12] Cobb S. R. Buhl E. H. Halasy K. Paulsen O. Somogyi P. (1995). Synchronization of neuronal activity in hippocampus by individual GABAergic interneurons. *Nature* 378 75–78. 10.1038/378075a0 7477292

[B13] Coote E. J. Rees A. (2008). The distribution of nitric oxide synthase in the inferior colliculus of guinea pig. *Neuroscience* 154 218–225. 10.1016/j.neuroscience.2008.02.030 18400412

[B14] de Bono M. Maricq A. V. (2005). Neuronal substrates of complex behaviors in C-elegans. *Annu. Rev. Neurosci.* 28 451–501. 10.1146/annurev.neuro.27.070203.144259 16022603

[B15] Ehret G. Schreiner C. E. (2005). “Spectral and intensity coding in the auditory midbrain,” in *The Inferior Colliculus*, eds Winer J. A. Schreiner C. E. (New York, NY: Springer), 312–345. 10.1007/0-387-27083-3_11

[B16] Fishell G. Rudy B. (2011). Mechanisms of inhibition within the telencephalon: “Where the wild things are”. *Annu. Rev. Neurosci.* 34 535–567. 10.1146/annurev-neuro-061010-113717 21469958 PMC3556485

[B17] FitzPatrick K. A. (1975). Cellular architecture and topographic organization of the inferior colliculus of the squirrel monkey. *J. Comp. Neurol.* 164 185–207. 10.1002/cne.901640204 810498

[B18] Fredrich M. Reisch A. Illing R. B. (2009). Neuronal subtype identity in the rat auditory brainstem as defined by molecular profile and axonal projection. *Exp. Brain Res.* 195 241–260. 10.1007/s00221-009-1776-7 19340418

[B19] Fujimoto H. Konno K. Watanabe M. Jinno S. (2016). Late postnatal shifts of parvalbumin and nitric oxide synthase expression within the GABAergic and glutamatergic phenotypes of inferior colliculus neurons. *J. Comp. Neurol.* 525 868–884. 10.1002/cne.24104 27560447

[B20] Goyer D. Silveira M. A. George A. P. Beebe N. L. Edelbrock R. M. Malinski P. T. (2019). A novel class of inferior colliculus principal neurons labeled in vasoactive intestinal peptide-Cre mice. *eLife* 8:e43770. 10.7554/eLife.43770 30998185 PMC6516826

[B21] Henkel C. K. Fuentes-Santamaria V. Alvarado J. C. Brunso-Bechtold J. K. (2003). Quantitative measurement of afferent layers in the ferret inferior colliculus: DNLL projections to sublayers. *Hear. Res.* 177 32–42. 10.1016/s0378-5955(02)00794-3 12618315

[B22] Irvine D. R. F. (1986). *The Auditory Brainstem.* Berlin: Springer-Verlag.

[B23] Ito T. (2020). Different coding strategy of sound information between GABAergic and glutamatergic neurons in the auditory midbrain. *J. Physiol.* 598 1039–1072. 10.1113/JP279296 31943205

[B24] Ito T. Bishop D. C. Oliver D. L. (2011). Expression of glutamate and inhibitory amino acid vesicular transporters in the rodent auditory brainstem. *J. Comp. Neurol.* 519 316–340. 10.1002/cne.22521 21165977 PMC3092437

[B25] Ito T. Bishop D. C. Oliver D. L. (2016). Functional organization of the local circuit in the inferior colliculus. *Anat. Sci. Int.* 91 22–34. 10.1007/s12565-015-0308-8 26497006 PMC4846595

[B26] Ito T. Hioki H. Sohn J. Okamoto S. Kaneko T. Iino S. (2015). Convergence of lemniscal and local excitatory inputs on large GABAergic tectothalamic neurons. *J. Comp. Neurol.* 523 2277–2296. 10.1002/cne.23789 25879870 PMC5446767

[B27] Ito T. Oliver D. L. (2014). Local and commissural IC neurons make axosomatic inputs on large GABAergic tectothalamic neurons. *J. Comp. Neurol.* 522 3539–3554. 10.1002/cne.23623 24796971 PMC4139440

[B28] Jones E. G. Hendry S. H. C. (1984). “Basket cells,” in *Cerebral Cortex: Cellular Components of the Cerebral Cortex*, eds Peters A. Jones E. G. (New York, NY: Plenum Press), 309–334.

[B29] Kawaguchi Y. Kubota Y. (1997). GABAergic cell subtypes and their synaptic connections in rat frontal cortex. *Cereb. Cortex* 7 476–486. 10.1093/cercor/7.6.476 9276173

[B30] King A. J. Palmer A. R. (1983). Cells responsive to free-field auditory stimuli in guinea-pig superior colliculus: distribution and response properties. *J. Physiol.* 342 361–381. 10.1113/jphysiol.1983.sp014856 6631739 PMC1193964

[B31] Kreeger L. J. Connelly C. J. Mehta P. Zemelman B. V. Golding N. L. (2021). Excitatory cholecystokinin neurons of the midbrain integrate diverse temporal responses and drive auditory thalamic subdomains. *Proc. Natl. Acad. Sci. U.S.A.* 118:e2007724118. 10.1073/pnas.2007724118 33658359 PMC7958253

[B32] Le Beau F. E. Rees A. Malmierca M. S. (1996). Contribution of GABA- and glycine-mediated inhibition to the monaural temporal response properties of neurons in the inferior colliculus. *J. Neurophysiol.* 75 902–919. 10.1152/jn.1996.75.2.902 8714663

[B33] LeBeau F. E. Malmierca M. S. Rees A. (2001). Iontophoresis *in vivo* demonstrates a key role for GABA(A) and glycinergic inhibition in shaping frequency response areas in the inferior colliculus of guinea pig. *J. Neurosci.* 21 7303–7312. 10.1523/jneurosci.21-18-07303.2001 11549740 PMC6762982

[B34] Lin F. G. Liu R. C. (2010). Subset of thin spike cortical neurons preserve the peripheral encoding of stimulus onsets. *J. Neurophysiol.* 104 3588–3599. 10.1152/jn.00295.2010 20943946 PMC3007649

[B35] Liu L. F. Palmer A. R. Wallace M. N. (2006). Phase-locked responses to pure tones in the inferior colliculus. *J. Neurophysiol.* 95 1926–1935. 10.1152/jn.00497.2005 16339005

[B36] Loftus W. C. Bishop D. C. Oliver D. L. (2010). Differential patterns of inputs create functional zones in central nucleus of inferior Colliculus. *J. Neurosci.* 30 13396–13408. 10.1523/Jneurosci.0338-10.2010 20926666 PMC2966845

[B37] Malmierca M. S. Blackstad T. W. Osen K. K. (2011). Computer-assisted 3-D reconstructions of Golgi-impregnated neurons in the cortical regions of the inferior colliculus of rat. *Hear. Res.* 274 13–26. 10.1016/j.heares.2010.06.011 20600744

[B38] Malmierca M. S. Blackstad T. W. Osen K. K. Karagulle T. Molowny R. L. (1993). The central nucleus of the inferior colliculus in rat: a Golgi and computer reconstruction study of neuronal and laminar structure. *J. Comp. Neurol.* 333 1–27. 10.1002/cne.903330102 7688006

[B39] Malmierca M. S. Izquierdo M. A. Cristaudo S. Hernandez O. Perez-Gonzalez D. Covey E. (2008). A discontinuous tonotopic organization in the inferior colliculus of the rat. *J. Neurosci.* 28 4767–4776. 10.1523/JNEUROSCI.0238-08.2008 18448653 PMC2440588

[B40] Malmierca M. S. Rees A. Le Beau F. E. Bjaalie J. G. (1995). Laminar organization of frequency-defined local axons within and between the inferior colliculi of the guinea pig. *J. Comp. Neurol.* 357 124–144. 10.1002/cne.903570112 7673462

[B41] Malmierca M. S. Saint Marie R. L. Merchan M. A. Oliver D. L. (2005). Laminar inputs from dorsal cochlear nucleus and ventral cochlear nucleus to the central nucleus of the inferior colliculus: two patterns of convergence. *Neuroscience* 136 883–894. 10.1016/j.neuroscience.2005.04.040 16344158

[B42] Martin R. L. Webster W. R. Serviere J. (1988). The frequency organization of the inferior colliculus of the guinea pig: a [14C]-2-deoxyglucose study. *Hear. Res.* 33 245–255. 10.1016/0378-5955(88)90155-4 3384759

[B43] McAlpine D. Jiang D. Palmer A. R. (1996). Interaural delay sensitivity and the classification of low best-frequency binaural responses in the inferior colliculus of the guinea pig. *Hear. Res.* 97 136–152. 10.1016/s0378-5955(96)80015-3 8844194

[B44] McCormick D. A. Connors B. W. Lighthall J. W. Prince D. A. (1985). Comparative electrophysiology of pyramidal and sparsely spiny stellate neurons of the neocortex. *J. Neurophysiol.* 54 782–806. 10.1152/jn.1985.54.4.782 2999347

[B45] Merchan M. Aguilar L. A. Lopez-Poveda E. A. Malmierca M. S. (2005). The inferior colliculus of the rat: quantitative immunocytochemical study of GABA and glycine. *Neuroscience* 136 907–925. 10.1016/j.neuroscience.2004.12.030 16344160

[B46] Morest D. K. (1964). The laminar structure of the inferior colliculus of the cat. *Anat. Rec.* 148:314.

[B47] Nakagawa H. Ikeda M. Houtani T. Ueyama T. Baba K. Kondoh A. (1995). Immunohistochemical evidence for enkephalin and neuropeptide Y in rat inferior colliculus neurons that provide ascending or commissural fibers. *Brain Res.* 690 236–240. 10.1016/0006-8993(95)00593-f 8535842

[B48] Nakamoto K. T. Mellott J. G. Killius J. Storey-Workley M. E. Sowick C. S. Schofield B. R. (2014). Ultrastructural characterization of GABAergic and excitatory synapses in the inferior colliculus. *Front. Neuroanat.* 8:108. 10.3389/Fnana.2014.00108 25400551 PMC4212260

[B49] Nelken I. (2008). Processing of complex sounds in the auditory system. *Curr. Opin. Neurobiol.* 18 413–417. 10.1016/j.conb.2008.08.014 18805485

[B50] Oliver D. L. (1987). Projections to the inferior colliculus from the anteroventral cochlear nucleus in the cat: possible substrates for binaural interaction. *J. Comp. Neurol.* 264 24–46. 10.1002/cne.902640104 2445792

[B51] Oliver D. L. Beckius G. E. Bishop D. C. Kuwada S. (1997). Simultaneous anterograde labeling of axonal layers from lateral superior olive and dorsal cochlear nucleus in the inferior colliculus of cat. *J. Comp. Neurol.* 382 215–229. 10.1002/(sici)1096-9861(19970602)382:2<215::aid-cne6>3.0.co;2-6 9183690

[B52] Oliver D. L. Kuwada S. Yin T. C. Haberly L. B. Henkel C. K. (1991). Dendritic and axonal morphology of HRP-injected neurons in the inferior colliculus of the cat. *J. Comp. Neurol.* 303 75–100. 10.1002/cne.903030108 2005240

[B53] Oliver D. L. Morest D. K. (1984). The central nucleus of the inferior colliculus in the cat. *J. Comp. Neurol.* 222 237–264. 10.1002/cne.902220207 6699209

[B54] Oliver D. L. Winer J. A. Beckius G. E. Saint Marie R. L. (1994). Morphology of GABAergic neurons in the inferior colliculus of the cat. *J. Comp. Neurol.* 340 27–42. 10.1002/cne.903400104 7909821

[B55] Olthof B. M. J. Gartside S. E. Rees A. (2019). Puncta of neuronal nitric oxide synthase (nNOS) mediate NMDA receptor signaling in the auditory midbrain. *J. Neurosci.* 39 876–887. 10.1523/JNEUROSCI.1918-18.2018 30530507 PMC6382984

[B56] Ono M. Yanagawa Y. Koyano K. (2005). GABAergic neurons in inferior colliculus of the GAD67-GFP knock-in mouse: electrophysiological and morphological properties. *Neurosci. Res.* 51 475–492. 10.1016/j.neures.2004.12.019 15740810

[B57] Palmer A. R. Shackleton T. M. Sumner C. J. Zobay O. Rees A. (2013). Classification of frequency response areas in the inferior colliculus reveals continua not discrete classes. *J. Physiol.* 591 4003–4025. 10.1113/jphysiol.2013.255943 23753527 PMC3764642

[B58] Palmer A. R. Wallace M. N. Arnott R. H. Shackleton T. M. (2003). Morphology of physiologically characterised ventral cochlear nucleus stellate cells. *Exp. Brain Res.* 153 418–426. 10.1007/s00221-003-1602-6 12955380

[B59] Perez-Gonzalez D. Malmierca M. S. Covey E. (2005). Novelty detector neurons in the mammalian auditory midbrain. *Eur. J. Neurosci.* 22 2879–2885. 10.1111/j.1460-9568.2005.04472.x 16324123

[B60] Peruzzi D. Sivaramakrishnan S. Oliver D. L. (2000). Identification of cell types in brain slices of the inferior colliculus. *Neuroscience* 101 403–416. 10.1016/s0306-4522(00)00382-1 11074163

[B61] Pinault D. (1996). A novel single-cell staining procedure performed *in vivo* under electrophysiological control: morpho-functional features of juxtacellularly labelled thalamic cells and other central neurons with biocytin or Neurobiotin. *J. Neurosci. Methods* 65 113–136. 10.1016/0165-0270(95)00144-1 8740589

[B62] Pollak G. D. (2013). The dominant role of inhibition in creating response selectivities for communication calls in the brainstem auditory system. *Hear. Res.* 305 86–101. 10.1016/j.heares.2013.03.001 23545427 PMC3778109

[B63] Ramon y Cajal S. (1995). “Histology of the nervous system of man and vertebrates,” in *The Inferior Colliculus*, eds Swanson N. Swanson L. W. (Oxford: Oxford University Press).

[B64] Rapisarda C. Bacchelli B. (1977). The brain of the guinea pig in stereotaxic coordinates. *Arch. Sci. Biol.* 61 1–37. 10.1007/978-1-4419-8372-5_1 400095

[B65] Rees A. Sarbaz A. Malmierca M. S. LeBeau F. E. N. (1997). Regularity of firing of neurons in the inferior colliculus. *J. Neurophysiol.* 77 2945–2965. 10.1152/jn.1997.77.6.2945 9212248

[B66] Rhode W. S. Oertel D. Smith P. H. (1983). Physiological response properties of cells labeled intracellularly with horseradish peroxidase in cat ventral cochlear nucleus. *J. Comp. Neurol.* 213 448–463. 10.1002/cne.902130408 6300200

[B67] Ribak C. E. (1992). Local circuitry of GABAergic basket cells in the dentate gyrus. *Epilepsy Res. Suppl.* 7 29–47. 1334668

[B68] Roberts R. C. Ribak C. E. (1987). An electron microscopic study of GABAergic neurons and terminals in the central nucleus of the inferior colliculus of the rat. *J. Neurocytol.* 16 333–345. 10.1007/bf01611345 3302119

[B69] Rockel A. J. Jones E. G. (1973). The neuronal organization of the inferior colliculus of the adult cat. I. The central nucleus. *J. Comp. Neurol.* 147 11–60. 10.1002/cne.901470103 4682181

[B70] Saldana E. Merchan M. A. (1992). Intrinsic and commissural connections of the rat inferior colliculus. *J. Comp. Neurol.* 319 417–437. 10.1002/cne.903190308 1376335

[B71] Schreiner C. E. Langner G. (1997). Laminar fine structure of frequency organization in auditory midbrain. *Nature* 388 383–386. 10.1038/41106 9237756

[B72] Shneiderman A. Henkel C. K. (1987). Banding of lateral superior olivary nucleus afferents in the inferior colliculus: a possible substrate for sensory integration. *J. Comp. Neurol.* 266 519–534. 10.1002/cne.902660406 2449472

[B73] Silveira M. A. Anair J. D. Beebe N. L. Mirjalili P. Schofield B. R. Roberts M. T. (2020). Neuropeptide Y expression defines a novel class of GABAergic projection neuron in the inferior colliculus. *J. Neurosci.* 40 4685–4699. 10.1523/JNEUROSCI.0420-20.2020 32376782 PMC7294802

[B74] Sturm J. Nguyen T. Kandler K. (2014). Development of intrinsic connectivity in the central nucleus of the mouse inferior Colliculus. *J. Neurosci.* 34 15032–15046. 10.1523/Jneurosci.2276-14.2014 25378168 PMC4220032

[B75] Syka J. Popelar J. Kvasnak E. Astl J. (2000). Response properties of neurons in the central nucleus and external and dorsal cortices of the inferior colliculus in guinea pig. *Exp. Brain Res.* 133 254–266. 10.1007/s002210000426 10968227

[B76] Wallace M. N. Palmer A. R. (2009). Functional subdivisions in low-frequency primary auditory cortex (AI). *Exp. Brain Res.* 194 395–408. 10.1007/s00221-009-1714-8 19205681

[B77] Wallace M. N. Shackleton T. M. Palmer A. R. (2012). Morphological and physiological characteristics of laminar cells in the central nucleus of the inferior colliculus. *Front. Neural Circuits* 6:55. 10.3389/fncir.2012.00055 22933991 PMC3422721

[B78] Wang Y. Gupta A. Toledo-Rodriguez M. Wu C. Z. Markram H. (2002). Anatomical, physiological, molecular and circuit properties of nest basket cells in the developing somatosensory cortex. *Cereb. Cortex* 12 395–410. 10.1093/cercor/12.4.395 11884355

[B79] West C. D. (1985). The relationship of the spiral turns of the cochlea and the length of the basilar membrane to the range of audible frequencies in ground dwelling mammals. *J. Acoust. Soc. Am.* 77 1091–1101. 10.1121/1.392227 3980863

[B80] Wynne B. Harvey A. R. Robertson D. Sirinathsinghji D. J. (1995). Neurotransmitter and neuromodulator systems of the rat inferior colliculus and auditory brainstem studied by in situ hybridization. *J. Chem. Neuroanat.* 9 289–300. 10.1016/0891-0618(95)00095-x 8719277

[B81] Wynne B. Robertson D. (1997). Somatostatin and substance P-like immunoreactivity in the auditory brainstem of the adult rat. *J. Chem. Neuroanat.* 12 259–266. 10.1016/s0891-0618(97)00219-6 9243345

[B82] Young E. D. Robert J. M. Shofner W. P. (1988). Regularity and latency of units in ventral cochlear nucleus - implications for unit classification and generation of response properties. *J. Neurophysiol.* 60 1–29. 10.1152/jn.1988.60.1.1 3404211

