# Supplementary figures and images for: Juxtacellular Labeling of Stellate, Disk and Basket Neurons in the Central Nucleus of the Guinea Pig Inferior Colliculus
Source: Front Neural Circuits. 2021 Nov 1;15:721015. doi: 10.3389/fncir.2021.721015 (PMC8592287; doi:10.3389/fncir.2021.721015)

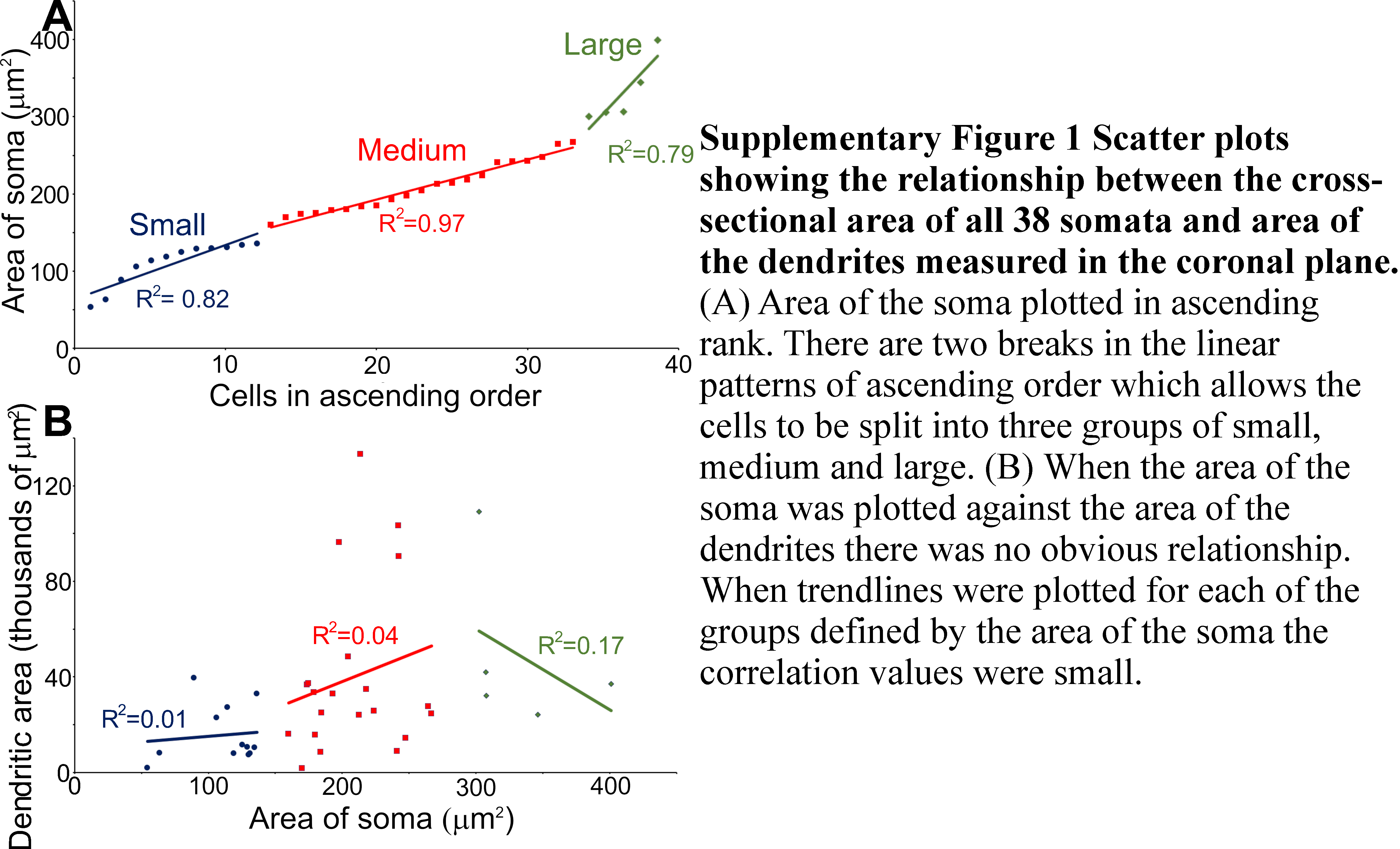

Supplement: Supplementary file 1 [file Data_Sheet_1.ZIP › Supplementary_Fig1.tif]

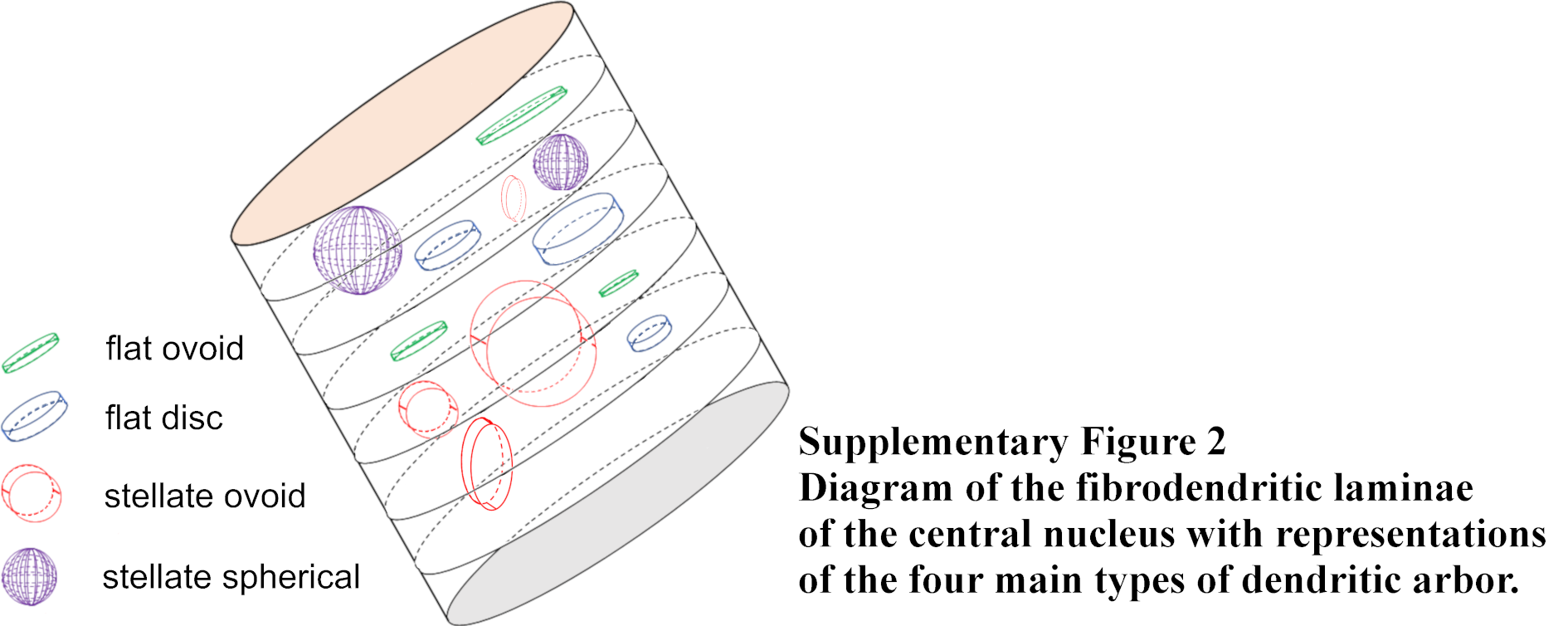

Supplement: Supplementary file 1 [file Data_Sheet_1.ZIP › Supplementary_Fig2.tif]

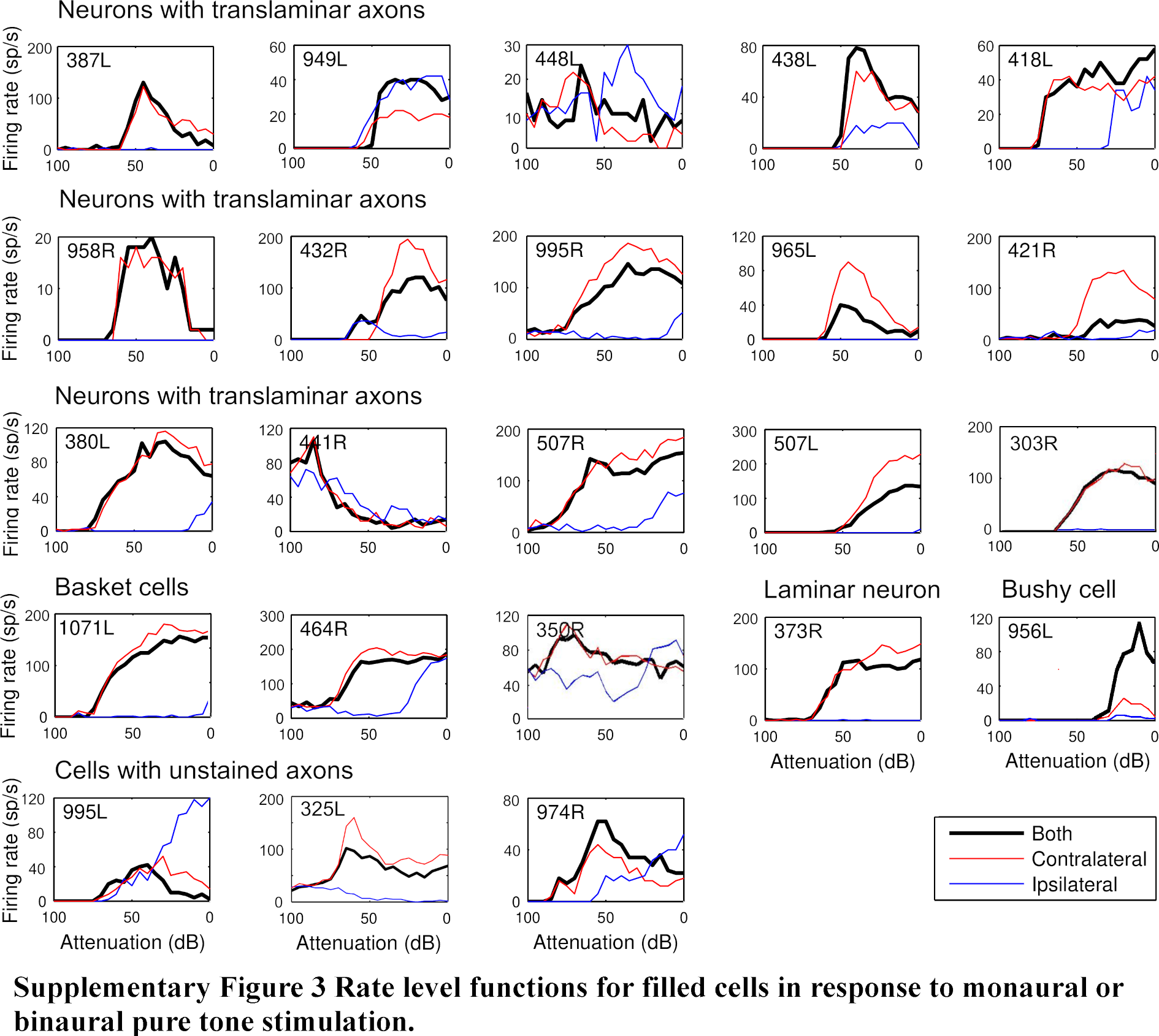

Supplement: Supplementary file 1 [file Data_Sheet_1.ZIP › Supplementary_Fig3.tif]

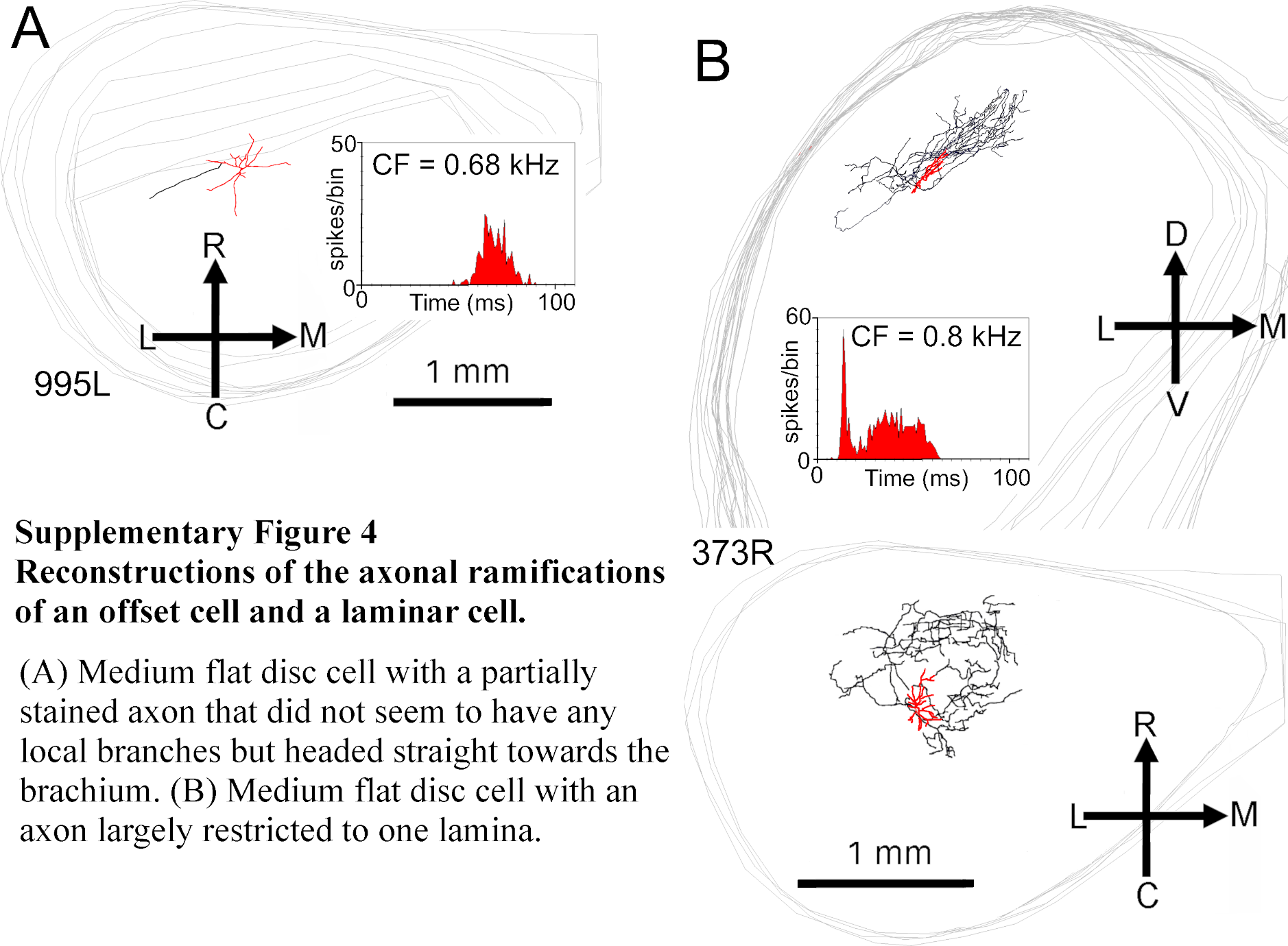

Supplement: Supplementary file 1 [file Data_Sheet_1.ZIP › Supplementary_Fig4.tif]

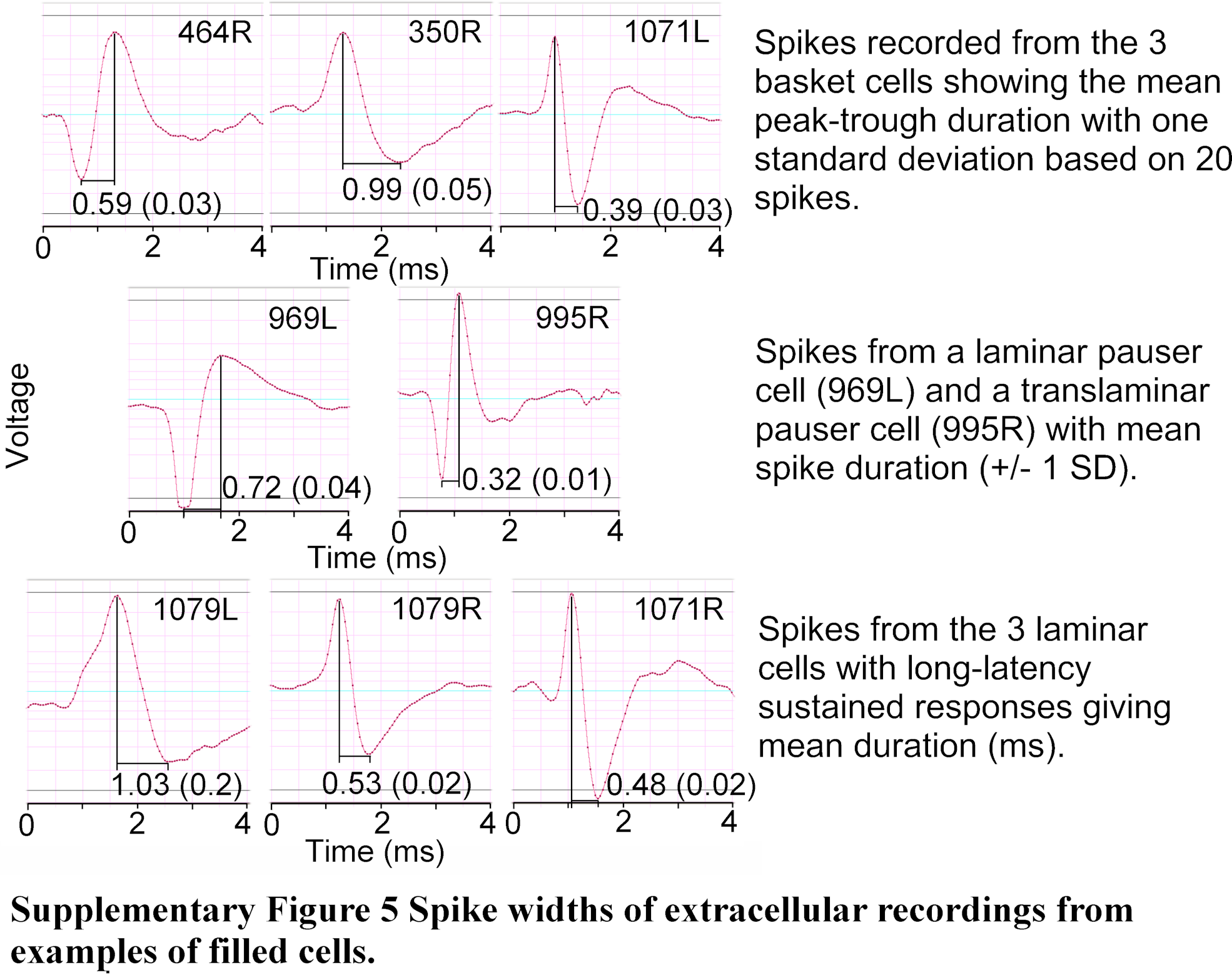

Supplement: Supplementary file 1 [file Data_Sheet_1.ZIP › Supplementary_Fig5.tif]

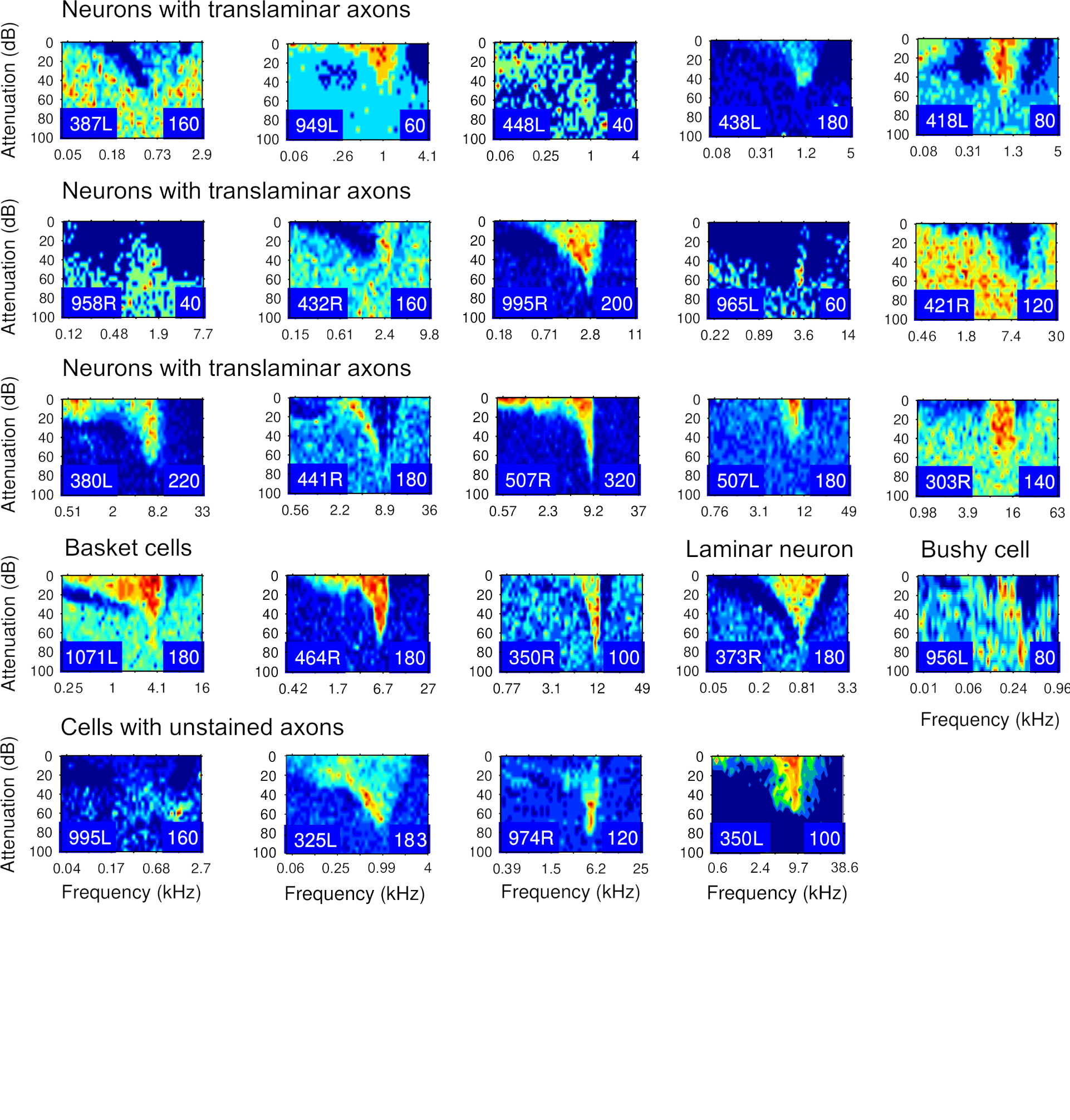

Supplement: Supplementary file 1 [file Data_Sheet_1.ZIP › Supplementary_Fig6.tif]

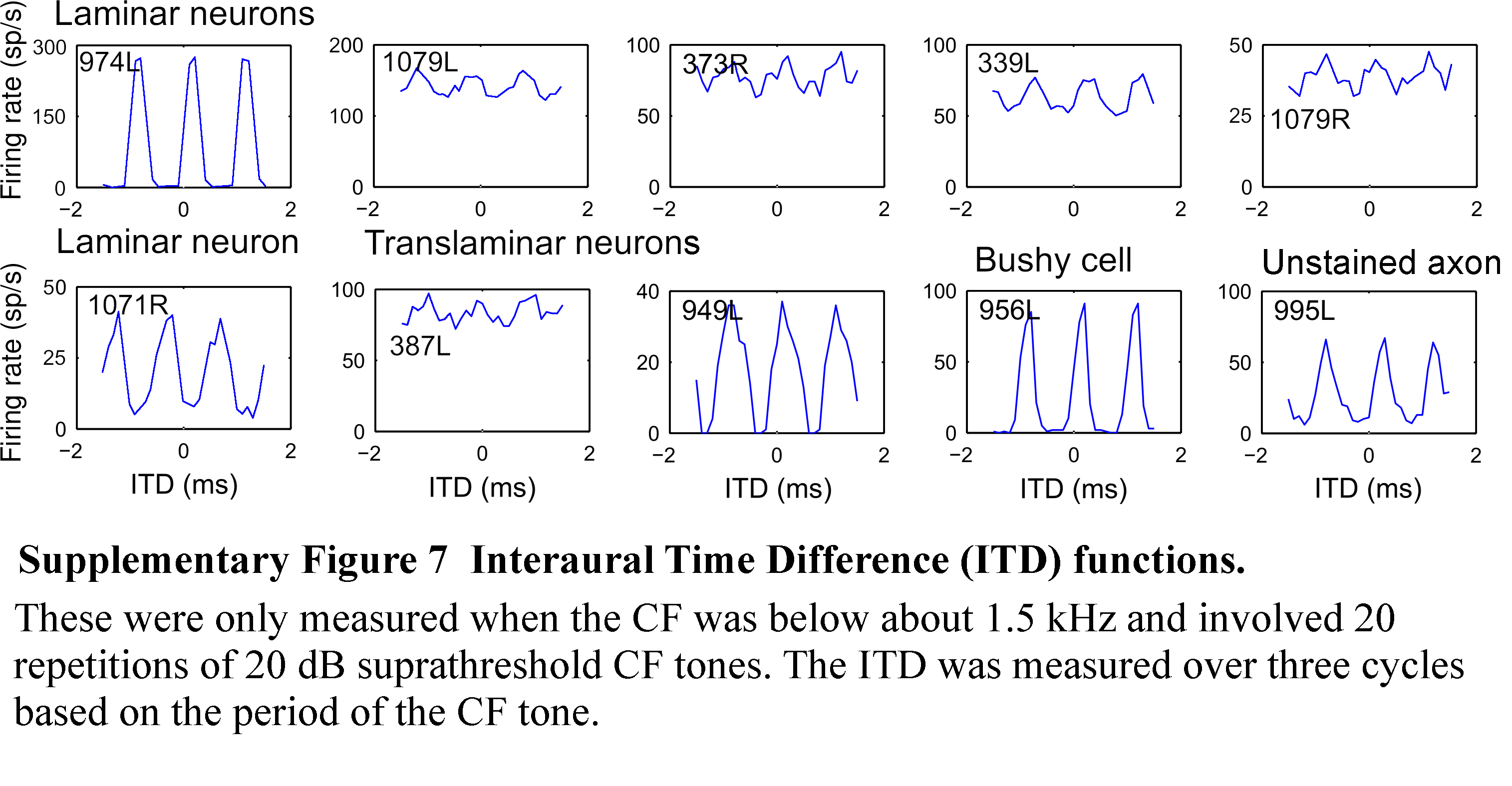

Supplement: Supplementary file 1 [file Data_Sheet_1.ZIP › Supplementary_Fig7.tif]

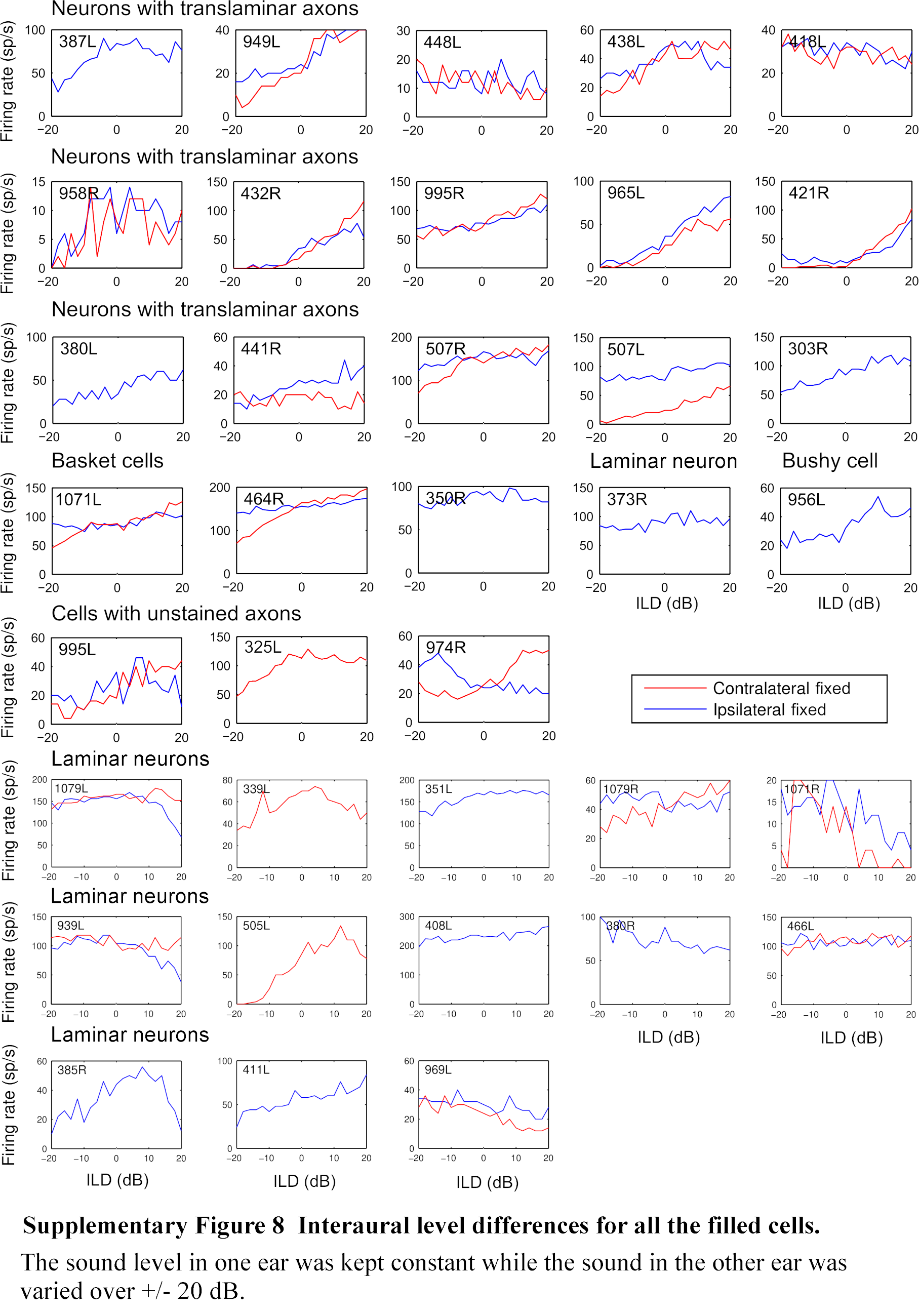

Supplement: Supplementary file 1 [file Data_Sheet_1.ZIP › Supplementary_Fig8.tif]
